# Core gene-based molecular detection and identification of *Acanthamoeba* species

**DOI:** 10.1038/s41598-020-57998-5

**Published:** 2020-01-31

**Authors:** Nisrine Chelkha, Priscilla Jardot, Iness Moussaoui, Anthony Levasseur, Bernard La Scola, Philippe Colson

**Affiliations:** 1 Aix-Marseille Univ., Institut de Recherche pour le Développement (IRD), Assistance Publique - Hôpitaux de Marseille (AP-HM), Microbes Evolution Phylogeny and Infections (MEPHI), 19-21 boulevard Jean Moulin, 13005 Marseille, France; 2 IHU Méditerranée Infection, 19-21 boulevard Jean Moulin, 13005 Marseille, France

**Keywords:** Water microbiology, Infectious diseases

## Abstract

*Acanthamoeba* spp. are predominant free-living amoebae of water and soil. They have been used as tools for the isolation and culture of microbes that resist after their phagocytosis, such as *Legionella*-like bacteria, and, more recently giant viruses for which differences in permissiveness have been reported. However, problems have been reported regarding their identification at the species level. The present work implemented specific PCR systems for the detection and identification of *Acanthamoeba* species through comparison of sequences and phylogenetic analyses. Thirty-three *Acanthamoeba* isolates were studied, including 20 reference strains and 13 isolates retrieved from water, soil or clinical samples. Previous delineation of a core genome encompassing 826 genes based on draft genome sequences from 14 *Acanthamoeba* species allowed designing PCR systems for one of these core genes that encodes an alanine-tRNA ligase. These primers allowed an efficient and specific screening to detect *Acanthamoeba* presence. In addition, they identified all 20 reference strains, while partial and complete sequences coding for 18S ribosomal RNA identified only 11 (55%). We found that four isolates may be considered as new *Acanthamoeba* species. Consistent with previous studies, we demonstrated that some *Acanthamoeba* isolates were incorrectly assigned to species using the 18S rDNA sequences. Our implemented tool may help determining which *Acanthamoeba* strains are the most efficient for the isolation of associated microorganisms.

## Introduction

*Acanthamoeba* spp. are ubiquitous in soil and water environments, and they play an important ecological role in the dynamics and functioning of terrestrial and aquatic ecosystems^1,2^. They have also been isolated in humans from cerebrospinal fluid, cornea, skin, nasal cavities, throat and digestive tract, as well as from other mammals and plants^3,4^. These amoebae have a free lifestyle and are characterized by two forms depending on external conditions: a trophozoite form corresponding to the active phase and a cystic form^5^.

*Acanthamoeba* spp. can be the hosts of several microorganisms that can survive post-phagocytosis, including bacteria and fungi, as well as giant viruses. Therefore, they can act as a reservoir and/or a vector for such microorganisms that encompass intracellular human pathogens, among which are *Legionella pneumophila* and *Mycobacterium* spp.^6,7^. *Acanthamoeba* spp. are themselves causative agents of diseases in humans, mostly in immunocompromised patients, being responsible for amoebic encephalitis granulomatosis that is potentially life-threatening, as well as for keratitis, sinusitis and skin lesions^2,7,8^.

Moreover, *Acanthamoeba* spp. have been used as tools to isolate amoeba-resistant microorganisms, primarily *Legionella*-like pathogens, and this led fortuitously to discover giant viruses of amoebae^9^. Giant viruses are characterized by a virion larger than 0.2 μm in size, which makes them visible by light microscopy^9–11^. They have gene repertoires that are far broader than those of other “classical” viruses, and their genomes notably encode translation components^12–14^. As several families of giant viruses were increasingly detected by culturing on *Acanthamoeba* spp., differences were observed between *Acanthamoeba castellanii* and *Acanthamoeba polyphaga*, the two most used species, regarding their susceptibility to giant viruses^15^. *A. castellanii* was demonstrated to be permissive to pandoraviruses and pithoviruses, *A. polyphaga* appeared more specifically permissive to mimiviruses^14,16,17^. Thus, in a study that searched for giant viruses in multiple environmental samples, different giant viruses were isolated using different amoebal species as culture support^15^.

Hence, the identification of *Acanthamoeba* species is essential for diagnosis purposes in clinical investigations and to discover giant viruses in research. Nevertheless, identifying *Acanthamoeba* species remains difficult. In one of the first studies that aimed at implementing a system for the identification of *Acanthamoeba* at the species level, 20 species were identified based on morphological criteria including the size and shape of the cysts^18^. However, this identification method has limitations. Indeed, cyst morphology can change depending on the culture conditions and can be highly variable for the same strain. Thus, this identification strategy needs to be supplemented by immunological, biochemical and physiological criteria for improved accuracy^19^. Molecular biology techniques have improved the identification of *Acanthamoeba* species. Thus, sequence analysis of 18S ribosomal DNA (rDNA) enabled distinguishing 20 genotypes, named T1 to T20, T4 genotype being the most common and related to human infections^20^. This approach used 18S rDNA gene sequences larger than 2,000 base pairs (bp), and it is considered that at least 90% of the full length of this gene needs to be analysed by phylogeny to reliably identify a species^21,22^. Nonetheless, *Acanthamoeba* isolates have been often identified based on 18S rDNA fragments shorter than 500 bp. In addition, the genomes of at least some *Acanthamoeba* species are polyploids, and nucleotide divergence between chromosomes has been estimated to be 2.5% although its precise level remains unknown^23,24^. In a preliminary work, we observed that 18S rDNA was present in multiple non-identical copies in *Acanthamoeba* genomes (unpublished data), which represents a pitfall for an accurate identification of these amoebal species. Therefore, we took advantage of the recent availability of a draft genome sequence for 14 *Acanthamoeba* species to seek to implement a reliable molecular system based on a conserved gene for the identification of *Acanthamoeba* species.

## Materials and Methods

### Draft genome sequence of 14 *Acanthamoeba* species

The draft genome sequences of 14 *Acanthamoeba* species publicly available on the NCBI website (http://www.ncbi.nlm.nih.gov/bioproject/; accession: PRJEB7687; Supplementary Table S1) were downloaded. They were part of the project «Phylogenomics of *Acanthamoeba* species» (Institute of Integrative Biology, University of Liverpool). We previously determined that these draft genome sequences contained between 24,098 and 224,482 scaffolds, corresponding to estimated lengths ranging from 55.6 to 120.6 megabp (Mbp)^25^.

### *Acanthamoeba* isolates and environmental and clinical samples

A total of 33 isolates of amoebae of the genus *Acanthamoeba* were tested. This encompassed 20 *Acanthamoeba* reference strains, of which 12 were strains whose draft genome sequences were available in the NCBI GenBank sequence database. They were ordered from the DSMZ biological resource center (https://www.dsmz.de/). No strain was available for *A. castellanii* ATCC 50370 or *A. pearcei*. Thirteen additional *Acanthamoeba* strains were isolated in our laboratory from environmental or clinical samples, in 5 and 8 cases, respectively (Table 1).

**Table 1:** *Acanthamoeba* species tested using the identification system, including reference strains and isolates from environmental and clinical samples.

Species	Strain	ATCC no. or reference	Isolation source	WGS project accession number
*Acanthamoeba castellanii*	Neff	30010	Soil, Pacific Grove, CA, 1957	AHJI01
*Acanthamoeba polyphaga*	CCAP 1501/3b	30872	Freshwater, Tuskegee, AL, 1965	CDFK01
	Linc-AP1	—	TJ Rowbotham	LQHA01
*Acanthamoeba healyi*	OC-3A	30866	St Martin’s River, MD, 1977	CDFA01
*Acanthamoeba mauritaniensis*	clone 1652	50253	Derived from the type strain, 1989	CDFE01 (Strain ATCC 50253)
*Acanthamoeba quina*	clone Vil3	50241	Derived from the type strain, 1989	CDFN01
*Acanthamoeba lugdunensis*	clone L3a	50240	Derived from an existing strain	CDFB01
*Acanthamoeba divionensis*	clone AA2	50238	Derived from the type strain, 1989	CDFI01
*Acanthamoeba rhysodes*	Haas	50368	Human eye infection, Houston, TX, 1985	CDFC01 (Strain ATCC 30973)
*Acanthamoeba royreba*	Oak Ridge	30884	BeWo human choriocarcinoma cells, Oak Ridge, TN, 1975	CDEZ01
*Acanthamoeba palestinensis*	CCAP 1547/1	30870	Soil, Israel, 1933	CDFD01
*Acanthamoeba astronyxis*	Ray & Hayes	30137	Soil, California, 1944	CDFH01
*Acanthamoeba lenticulata*	E18-2	50690	Derived from strain CDC:V023 (eye of an adult human female with keratitis); sediment from 40 mile Philadelphia dump site, SE edge of sewage site	CDFG01 (Strain ATCC 30841)
*Acanthamoeba culbertsoni*	Lilly A-1	30171	Primary monkey kidney tissue culture, India, 1957	CDFF01
*Acanthamoeba griffini*	TIO:H37	50702	Human *Acanthamoeba* keratitis, Glasgow, UK	—
*Acanthamoeba hatchetti*	2HH	PRA-113	Clinical specimen - human, Vienna Austria, 1996	—
*Acanthamoeba terricola*	—	30134	Arable soil, Seine-et-Oise, France, 1960	—
*Acanthamoeba tubiashi*	OC-15C	30867	St. Martin’s River, MD, 1978	—
*Acanthamoeba triangularis*	SH 621	50254	Derived from the type strain, 1989	—
*Acanthamoeba stevensoni*	RB-F-1-AX	50438	—	—
*Acanthamoeba* sp. clinical isolate 1	—	—	Clinical isolate	—
*Acanthamoeba* sp. clinical isolate 2	—	—	Clinical isolate	—
*Acanthamoeba* sp. clinical isolate 3	—	—	Clinical isolate	—
*Acanthamoeba* sp. clinical isolate 4	—	—	Clinical isolate	—
*Acanthamoeba* sp. clinical isolate 5	—	—	clinical isolate	—
*Acanthamoeba* sp. clinical isolate 6	—	—	Clinical isolate	—
*Acanthamoeba* sp. clinical isolate 7	—	—	Clinical isolate	—
*Acanthamoeba* sp. clinical isolate 8	—	—	Clinical isolate	
*Acanthamoeba* sp. environmental isolate 1	—	—	Environmental isolate	—
*Acanthamoeba* sp. environmental isolate 2	—	—	Environmental isolate	—
*Acanthamoeba* sp. environmental isolate 3	—	—	Environmental isolate	—
*Acanthamoeba* sp. environmental isolate 4	—	—	Environmental isolate	—
*Acanthamoeba* sp. environmental isolate 5	—	—	Environmental isolate	—

### Design of PCR primer systems

In a previous work, a prediction of ORFs was performed using the Prodigal program for the draft genome sequences of *A. polyphaga* ATCC 30872 and 13 other *Acanthamoeba* species^25,26^. A search for homologous sequences in the NCBI GenBank non redundant protein sequence database (nr) was then performed with the BLASTp program (https://blast.ncbi.nlm.nih.gov) using non-redundant scaffolds of *A. polyphaga* ATCC 30872 as queries. The sequences of genes larger than 300 nucleotides predicted from the draft genome sequences of the 14 *Acanthamoeba* species were used in the subsequent analyses that consisted in a BLASTp all-against-all search with protein sequences predicted for each of these genes^25^. We delineated the core genome of these amoebae and then extracted the nucleotide sequences of these genes that were found to be present in all 14 *Acanthamoeba* species. Thereafter, we performed with the BLASTn program systematic pairwise comparisons between these genes^27^. Only genes for which none of these pairwise comparisons resulted in 100% nucleotide identity, i.e., their sequence differed in each of the 14 draft genome sequences, were further examined. For each of these genes, nucleotide sequences were aligned using the MUSCLE software^28^. Thereafter, nucleotide alignements were screened using the SVARAP tool^29^ in order to identify areas (i) conserved in the sequences from the 14 different species, where universal PCR primers or probes could hybridize reliably, and (ii) that flank a region variable enough to enable sequence-based discrimination between *Acanthamoeba* species (i.e., with nucleotide identities < 99%). Additionaly, four primer systems were designed using the same method to target the complete 18S rDNA of reference strains *Acanthamoeba lugdunensis* ATCC 50240, *A. polyphaga* strain Linc-AP1, *Acanthamoeba terricola* ATCC 30134, *Acanthamoeba hatchetti* ATCC PRA-113, *Acanthamoeba griffini* ATCC 50702 and *Acanthamoeba stevensoni* ATCC 50438, as well as 18S rDNA of *Acanthamoeba* spp. isolates from environmental and clinical samples. Finally, we extracted the nucleotide sequences corresponding to the region amplified with primer system Ami6F1 (5′ CCAGCTCCAATAGCGTATATT 3′) and Ami9R (5′ GTTGAGTCGAATTAAGCCGC 3′) from the 18S rDNA complete sequence^30^. The complete and partial 18S rDNA sequences were considered to provide a reference identification, and were used for comparative analyses.

### PCR and sequencing of alanine-tRNA ligase

DNA extraction was performed using the EZ1 DNA tissue kit (Qiagen, CA, USA) with the bacteria card on the EZ1 instrument. *Acanthamoeba* DNA was amplified by PCR with three primer systems named Lig1, Lig2 and Lig3, and the AmpliTaq Gold 360 Master mix (Applied Biosystems, Foster City, CA, USA). PCR reaction mixture (25 µL per sample) was prepared as follows: DNA extract (2 µL), forward primer (1 µL, 10 µM; Eurogentec, Seraing, Belgium), reverse primer (1 µL, 10 µM, Eurogentec), master mix with dNTPs and Taq DNA polymerase (2X , 12.5 µL) and DEPC-treated water (8.5 µL). The PCR amplification program included an initial denaturation step at 95 °C for 10 min followed by 35 PCR cycles: each cycle consisted of a denaturation step at 95 °C for 30 s, an annealing step at 58–60 °C for 30 s and an extension step at 72 °C for 1 min. The program also included a final extension step at 72 °C for 7 min. PCR products were separated using electrophoresis in 1.5% agarose gel and were visualised with SYBR safe (Invitrogen, Carlsbad, CA, USA). The Nucleofast 96 PCR clean-up kit (Macherey Nagel, Düren, Germany) was used for the purification of PCR products according to the manufacturer’s instructions. DNA was sequenced using the Sanger sequencing method on an automatic sequencer (ABI-3130 XL genetic analyser; Applied Biosystems) with the BigDye Terminator v1.1 sequencing kit (Applied Biosystems). The sequencing data were analysed with the Chromas Pro 1.7.1 software (Technelysium Pty, Ltd., Tewantin, Queensland, Australia).

### Phylogenetic analyses

Sequence alignments were performed using the Muscle program, and phylogenetic trees were constructed with the MEGA software using a Neighbor-Joining method, with a Maximum Composite Likelihood substitution model (considering both transitional and transversional substitutions), uniform rates, homogeneous patterns among lineages and pairwise deletion of gaps^31^. The alanine-tRNA ligase sequence of the amoeba *Dictyostelium discoideum* strain AX4 available in the NCBI GenBank database (accession number: NC_007089.4) was used as an outgroup for these phylogenetic analyses. Overall, the identification of *Acanthamoeba* species was based on the two following criteria: nucleotide similarity <100% in pairwise comparisons and bootstrap threeshold <90% in phylogenetic analyses.

## Results

### Design of a universal PCR system based on the core gene set of *Acanthamoeba* species

Pairwise comparative analyses identified only 15 candidate genes among the 826 genes conserved in the core genome of all 14 *Acanthamoeba* species whose draft genome sequences were available and with nucleotide sequences divergence for each of these species (Supplementary Table S2). Out of these 15 genes, only one that was predicted to encode an alanine-tRNA ligase was suitable to design PCR systems based on our criteria. Indeed, it harbored regions with a high level of nucleotide identity in the 14 *Acanthamoeba* species, which flanked nucleotide sequences displaying sufficient nucleotide diversity in the different *Acanthamoeba* species to allow their identification.

Three “universal” PCR primer systems, named Lig1, Lig2 and Lig3, could be designed in different regions of this gene (Table 2; Supplementary Fig. S1). Nucleotide identity levels between these PCR primers and targeted regions differed for the different PCR systems. For the first system (Lig1), nucleotide identity for the forward primer ranged between 94% (for species *A. culbertsoni* ATCC 30171, *A. astronyxis* ATCC 30137 and *A. divionensis* ATCC 50238) and 100%, and for the reverse primer it ranged between 94% (for *A. culbertsoni*, *A. astronyxis*, *A. divionensis* and *A. lenticulata* ATCC 50690) and 100% (for the other *Acanthamoeba* species). For the second system (Lig2), nucleotide identity for the forward primer was of 100% for the 14 *Acanthamoeba* species and ranged between 89% (for *A. astronyxis* and *A. divionensis*) and 100% for the reverse primer. For the third system (Lig3), nucleotide identity for the forward primer ranged between 93% (for species *A. culbetsoni*, *A. astronyxis* and *A. divionensis*) and 100%, while the reverse primer had a nucleotide identity of 100% in all cases with its targeted regions. Regarding the amplicons generated using the first system (Lig1), the draft genome sequences of the 14 *Acanthamoeba* species have nucleotide identities that range in pairwise comparisons between 67% and 100%. For the second system (Lig2), nucleotide identities in pairwise comparisons range between 45% and 100%. For the third system (Lig3), nucleotide identities range in pairwise comparisons between 55% and 100% (Supplementary Table S3).

**Table 2:** Set of primers designed for the alanine-tRNA ligase gene from *Acanthamoeba* spp.

Primer name	Forward/Reverse	Sequence 5′-3′	Length (nucleotides)	Amplicon size (bp)
Lig1_F	Forward	CTTCAAGGAGGAGGCCAT	18	684 bp
Lig1_R	Reverse	CTGCTTGCCGTAKCGCAC	18	
Lig2_F	Forward	GAGAACTTCTGGGAGATGGG	20	783 bp
Lig2_R	Reverse	CCTTCTCCTCGGCCATGAG	19	
Lig3_F	Forward	CTCTGCGGTGGTACCCAC	18	472 bp
Lig3_R	Reverse	CGGATGGCCTTGATGGC	17	

bp, base pair.

### PCR detection and Sanger sequencing of the alanine-tRNA ligase encoding gene from *Acanthamoeba* strains

As assessed by migration on agarose gel, a PCR product was obtained for all *Acanthamoeba* reference strains and environmental or clinical isolates tested by conventional PCR systems Lig1 and Lig3 designed here. We obtained amplicons with the expected sizes of 684 and 472 bp, respectively. Using PCR system Lig2, a PCR product was obtained for all but one *Acanthamoeba* isolate, the reference strain *A. tubiashi* ATCC 30867*;* amplicon size was 783 bp.

Subsequently, Sanger sequencing of fragments of the alanine-tRNA ligase gene was successfully performed with the Lig1 system for 19 (95%) of the 20 reference strains of *Acanthamoeba* tested, the exception being *A. tubiashi*. Using the system Lig2, a sequence was obtained for 18 (90%) of the 20 *Acanthamoeba* reference strains. Failures occurred for *A. polyphaga* strain Linc-AP1 and *A. tubiashi*. Using the system Lig3, a sequence was obtained for all *Acanthamoeba* reference strains. When applied to the *Acanthamoeba* environmental and clinical isolates, Sanger sequencing using the system Lig1 was successful for 10 (77%) of the 13 isolates. Failures occurred for *Acanthamoeba* sp. clinical isolate 2, *Acanthamoeba* sp. environmental isolate 3 and *Acanthamoeba* sp. environmental isolate 5. Using system Lig2, a sequence was obtained for 11 (85%) of the 13 isolates. Failures occurred for *Acanthamoeba* sp. environmental isolate 3 and *Acanthamoeba* sp. environmental isolate 5. Using the Lig3 system, a sequence was obtained for all but one isolate, *Acanthamoeba* sp. environmental isolate 5. We retested twice the Sanger sequencing in case of failure to obtain a sequence, but failures were reproducible for all samples with all three systems.

### Identification of *Acanthamoeba* species based on phylogenetic analysis of alanine-tRNA ligase gene fragments

#### Acanthamoeba reference strains

**Figure 1:**
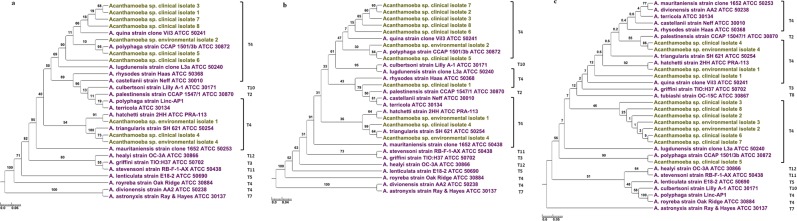
Phylogenetic trees for regions of the alanine-tRNA ligase encoding genes from the *Acanthamoeba* species targeted by primers Lig1 (**a**), primers Lig2 (**b**) and primers Lig3 (**c**). Five hundred bootstrap replicates were performed. The scale bars indicate the number of nucleotide substitutions per site. In purple: reference strains; in light green: environmental or clinical isolates.

**Figure 2:**
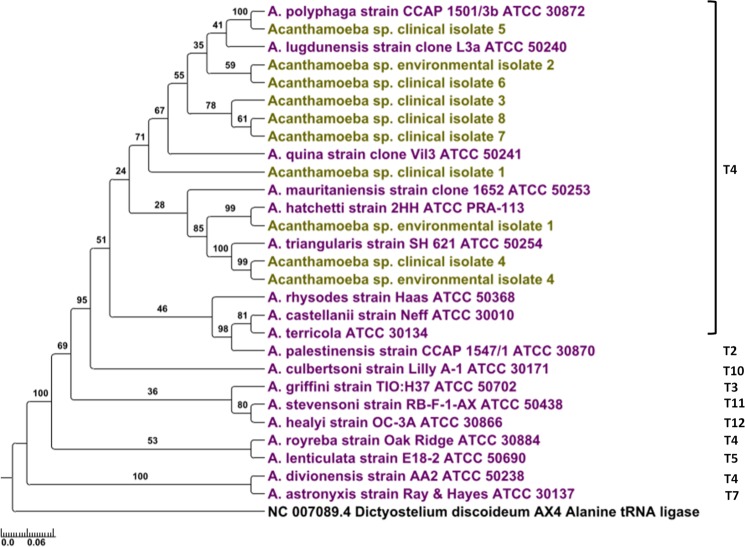
Phylogenetic tree for concatenated sequences of the alanine-tRNA ligase encoding genes from the *Acanthamoeba* species targeted by primers Lig1, Lig2 and Lig3. Five hundred bootstrap replicates were performed. The scale bars indicate the number of nucleotide substitutions per site. In purple: reference strains; in light green: environmental or clinical isolates.

In a next step, identification of *Acanthamoeba* species was considered accurate when sequences from reference strains could be differentiated between each other based on both a bootstrap value < 90% and a nucleotide identity <100%. Thus, the sequences considered as resulting from an *Acanthamoeba* previously identified at the species level had to be unique in our experiment. Phylogenetic reconstructions of the alanine-tRNA ligase gene fragments have shown that it is possible to identify 14 (74%) out of the 19 species using the Lig1system. Five out of the 19 reference strains, including *A. castellanii* strain Neff, *A. palestinensis* ATCC 30870 and *A. culbertsoni*, *A. astronyxis* and *A. divionensis* could not be discriminated using this system (Fig. 1a; Supplementary Table S4). System Lig2 made it possible to identify 12 (67%) out of the 18 species. This second system did not allow to discriminate reference strains of *A. castellanii* strain Neff*, A. terricola*, *A. culbertsoni, A. lugdunensis*, *A. astronyxis* and *A. divionensis* (Fig. 1b; Supplementary Table S4). Finally, system Lig3 enabled the identification of 16 (80%) out of the 20 species. This system did not distinguish between the reference strains *A. castellanii* strain Neff, *A. triangularis*, *A. polyphaga* strain Linc-AP1 and *A. royreba* (Fig. 1c; Supplementary Table S4). The phylogenetic reconstruction based on concatenated sequences of the three regions showed that it was possible to identify 13 (72%) out of the 18 species for which sequences from the three regions were successfully obtained by sequencing. Seven *Acanthamoeba* reference strains, namely *A. stevensoni*, *A. healyi* ATCC 30866*, A. griffini*, *A. lenticulata, A. royreba, A. divionensis* and *A. astronyxis*, sharply differed from the other strains regarding their phylogeny and sequence similarity (64% to 92%) (Fig. 2; Supplementary Table S4). Finally, the complementarity between the primer systems Lig3 and Lig2 made it possible to identify all the reference strains (Supplementary Fig. S2).

**Figure 3:**
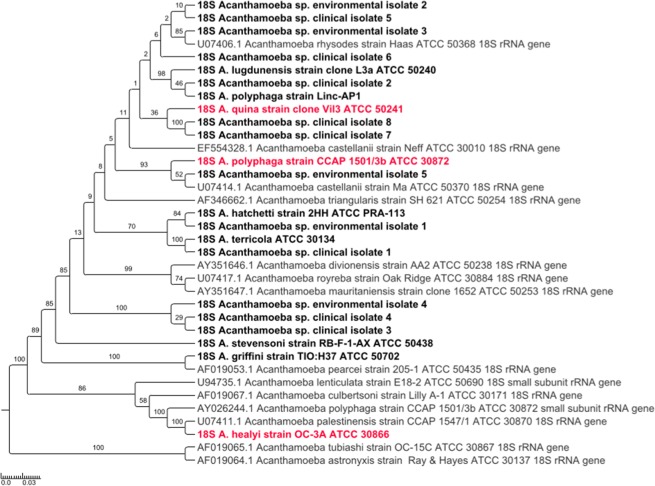
Phylogenetic tree for 18S ribosomal DNA partial sequence of *Acanthamoeba* species. Nucleotide sequences of the 18S ribosomal DNA were obtained using the primers Ami6F1/Ami9R. Five hundred bootstrap replicates were performed. The scale bars indicate the number of nucleotide substitutions per site. In gray: 18S ribosomal DNA sequences retrieved from the NCBI GenBank nucleotide sequence database for reference strains; in black: 18S ribosomal DNA sequences obtained using primers designed in the present study; in pink: 18S ribosomal DNA sequences retrieved from the draft genome sequences of *Acanthamoeba* species.

**Figure 4:**
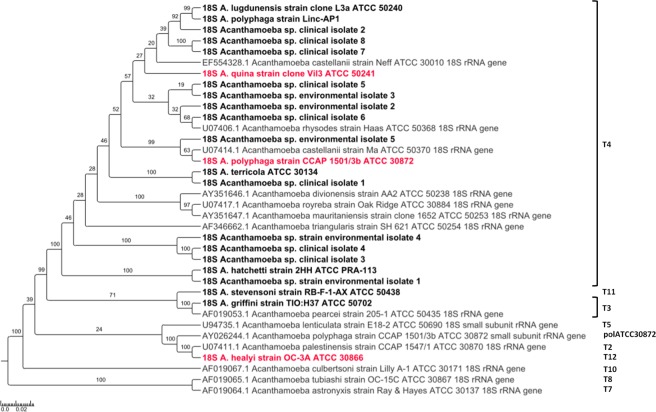
Phylogenetic tree for 18S ribosomal DNA of *Acanthamoeba* species. The genotypes based on previous studies or suggested here were represented for each species. Five hundred bootstrap replicates were performed. The scale bars indicate the number of nucleotide substitutions per site. In gray: 18S ribosomal DNA sequences retrieved from the NCBI GenBank nucleotide sequence database for reference strains; in black: 18S ribosomal DNA sequences obtained using primers designed in the present study; in pink: 18S ribosomal DNA sequences retrieved from the draft genome sequences of *Acanthamoeba* species.

Regarding phylogenetic analyses and sequence similarities based on the partial 18S rDNA sequence, they allowed identifying *Acanthamoeba* species for only 11 (55%) out of 20 strains (Fig. 3; Supplementary Table S4). Similarily, the complete 18S rDNA sequence enabled identifying 11 (55%) of the 20 strains (Fig. 4). Indeed, complete sequences were all different in pairwise comparison by at least one nucleotide (range: 51,8–99,9%), but phylogenetic analysis was less discriminant than the one based on the three alanine-tRNA ligase fragments. The classification of reference strains according to their genotypes was determined phylogenetically based on the complete 18S rDNA gene (Table 3; Fig. 4). Thirteen species formed a cluster with reference strains of genotype T4. They included strains *A. castellanii* strain Neff and *A. castellanii* ATCC 50370, and also *A. polyphaga* strain Linc-AP1 and the 18S rDNA sequence extracted from the draft genome sequence of *A.polyphaga* ATCC 30872. Both species *A. pearcei* and *A. griffini*, which belong to genotype T3, were clustered together. Additionally, the second 18S rDNA sequence available for the strain *A. polyphaga* ATCC 30872 (AY026244.1) appeared to differ from that extracted from the draft genome sequence of this strain, nor has it clustered with it in phylogenetic reconstruction or with any sequence from a described genotype (Fig. 4). Corsaro *et al*. suggested a new group for this strain, the polATCC30872^21^. Genotype clustering of *Acanthamoeba* reference strains was similar in both phylogenies based on concatenated alanine-tRNA ligase gene fragments and complete 18S rDNA sequence, except for *A. royreba* and *A. divionensis*. Both species did not form a cluster with reference strains of genotype T4 using the alanine-tRNA ligase gene fragments.

**Table 3:** Set of primers designed for 18S ribosomal DNA from *Acanthamoeba* spp.

Primer name	Forward/Reverse	Sequence 5′-3′	Length (nucleotides)	Amplicon size (bp)
Acant_18S_F1	Forward	TCATATGCTTGTCTCAAAGAT	21	700
Acant_18S_R1	Reverse	GCTTTTTAACTGCAACAACTT	21	
Acant_18S_F2	Forward	GCGGTAATTCCAGCTCCAAT	20	678
Acant_18S_R2	Reverse	TGGTGTTTTGTATTCAACGTC	21	
Acant_18S_F3	Forward	ACCATAAACGATGCCGACCA	20	700
Acant_18S_R3	Reverse	ACTCGTTGGATTAATCAGTGT	21	
Acant_18S_F4	Forward	CCTTAGATGTTCTGGGCCG	19	452
Acant_18S_R4	Reverse	GACCTTGTTACGACTTCTCC	20	

bp, base pair.

#### Acanthamoeba environmental and clinical isolates

*Acanthamoeba* environmental and clinical strains showed in some cases high sequence similarity of alanine-tRNA ligase gene fragments and concatenated sequences (99.2%-100%) with the reference strains and were phylogenetically close to them. Indeed, gene sequences obtained using system Lig1 of *Acanthamoeba* sp. clinical isolate 4 and *Acanthamoeba* sp. environmental isolate 4 showed a nucleotide identity of 100% with the reference species *A. triangularis*, and both isolates were clustered with this reference strain. This was also the case for *Acanthamoeba* sp. clinical isolate 5 with *A. polyphaga* ATCC 30872, and for *Acanthamoeba* sp. clinical isolates 7 and 8 with *A. quina*, while *Acanthamoeba* sp. environmental isolate 1 was clustered with *A. hatchetti* (Fig. 1a; Supplementary Table S4). Using system Lig2, a nucleotide identity of 100% was observed between *Acanthamoeba* sp. clinical isolate 6 and reference strain *A. quina*, and between *Acanthamoeba* sp. clinical isolate 4 and *Acanthamoeba* sp. environmental isolate 4 that were clustered with *A. triangularis* (Fig. 1b; Supplementary Table S4). Finally, system Lig3 showed that *Acanthamoeba* sp. clinical isolate 5 was clustered with *A. polyphaga* ATCC 30872 and that their gene sequences were identical at 100%. Furthermore, *Acanthamoeba* sp. environmental isolate 1 was clustered with *A. hatchetti*, and a nucleotide identity of 100% was observed between *Acanthamoeba* sp. clinical isolate 1 and reference strains *A. castellanii* strain Neff and *A. triangularis* (Fig. 1c; Supplementary Table S4). Phylogenetic analyses and sequence identities based on concatenated sequences of the three regions showed that *Acanthamoeba* sp. clinical isolate 5 was clustered with reference strain *A. polyphaga* ATCC 30872. Similarly, *Acanthamoeba* sp. environmental isolate 1 was clustered with *A. hatchetti*, and *Acanthamoeba* sp. clinical isolate 4 and *Acanthamoeba* sp. environmental isolate 4 were closely related to *A. triangularis* (Fig. 2; Supplementary Table S4).

Regarding phylogenetic analyses and sequence similarities based on both partial and complete 18S rDNA sequence, *Acanthamoeba* sp. clinical isolate 1 was clustered with *A. terricola*. Similar observations were obtained between *Acanthamoeba* sp. clinical isolate 2 and reference strains *A. polyphaga* strain Linc-AP1 and *A. lugdunensis*, and between *Acanthamoeba* sp. environmental isolate 5 and *A. polyphaga* ATCC 30872. In addition, *Acanthamoeba* sp. environmental isolate 1 was clustered with *A. hatchetti* based on the phylogeny of the complete 18S rDNA sequence (Fig. 3; Supplementary Table S4). Based on the complete 18S rDNA gene, all *Acanthamoeba* isolates were clustered with reference strains of genotype T4 (Fig. 4). As observed for reference strains, clustering of *Acanthamoeba* isolates was similar in phylogenies based on concatenated alanine-tRNA ligase gene fragments and complete 18S rDNA sequences.

Finally, phylogenetic analyses and sequence similarities for the three alanine-tRNA ligase gene fragments, their concatenation, and 18S partial and complete rDNA sequences allowed four environmental or clinical isolates to be assigned to the *Acanthamoeba* reference species. Indeed, *Acanthamoeba* sp. clinical isolate 5 seems to belong to species *A. polyphaga*, *Acanthamoeba* sp. environmental isolate 1 belongs to species *A. hatchetti* and *Acanthamoeba* sp. clinical isolate 4 and *Acanthamoeba* sp. environmental isolate 4 seem closely related to species *A. triangularis*. In contrast, we observed that, on the basis of congruent findings with the different PCR systems, *Acanthamoeba* sp. clinical isolate 8, *Acanthamoeba* sp. clinical isolate 7, *Acanthamoeba* sp. environmental isolate 2 and *Acanthamoeba* sp. environmental isolate 3 were genetically distant from the reference strains and may be considered as belonging to new species.

## Discussion

We have developed here PCR systems for the rapid identification of *Acanthamoeba* species that proved to be accurate for a large number of strains from various sources. Preliminary studies carried out on 14 draft genome sequences of different *Acanthamoeba* species have resulted in the selection of a target gene that encodes an alanine-tRNA ligase for the efficient identification of *Acanthamoeba* at the species level. Further analysis of the nucleotide diversity between sequences of this gene in the 14 species has led to the design of three universal PCR primer systems. The detection accuracy of one PCR system was demonstrated by the recovery of a sequence for all but one *Acanthamoeba* isolate. The performance of the PCR systems Lig1 and Lig2 was lower as they both failed to amplify four *Acanthamoeba* spp. This might be due to numerous and/or critical mismatches in regions where primers hybridize. The complementarity of the two primer systems Lig3 and Lig2 enabled identifying all *Acanthamoeba* reference strains, which was not the case with partial sequences of 18S rDNA (830 bp on average)^30^, and even when using the complete 18S rDNA sequences (2,165 bp on average). Based on sequences of the 18S rDNA complete gene, phylogeny performed here showed a clustering of reference strains from the same genotypes. Alternatively, primer system Lig1 may be used for the identification of other *Acanthamoeba* isolates in future studies.

Furthermore, our results suggest that some *Acanthamoeba* isolates were incorrectly assigned to species. Indeed, strains *A. castellanii* Neff and *A. castellanii* ATCC 50370 were not clustered together using either partial or complete 18S rDNA sequences, and this was also the case for strains *A. polyphaga* Linc-AP1 and *A. polyphaga* ATCC 30872. In addition, the partial and complete 18S rDNA of the strain *A. polyphaga* ATCC 30872 (AY026244.1) and that from the draft genome sequence of this strain were found to differ considerably. Taken together, these findings question the accuracy of the identification of *Acanthamoeba* species using either partial or complete 18S rDNA. Recent findings have shown that a second strain of *A. castellanii* ATCC 50370, which was not available during our study and therefore not tested here, did not cluster with *A. castellanii* strain Neff. This observation was based on phylogenetic analyses and the synteny in *Acanthamoeba* spp. draft genome sequences of genes with viral homologs as best hits^25^. Phylogenies also showed that *A. castellanii* ATCC 50370 was close to *A. polyphaga* ATCC 30872, thus highlighting a possible misidentification of these isolates.

Finally, our molecular systems implemented have made it possible to provide for the first time a classification for certain *Acanthamoeba* isolates. Thus, based on nucleotide differences and phylogenetic analyses of alanine-tRNA ligase fragments and concatenated sequences as well as partial and complete 18S rDNA sequences, at least four isolates may be considered as belonging to a new species, including two environmental isolates and two clinical isolates.

In summary, we have set up precise molecular systems to identify *Acanthamoeba* species, as an alternative to those based on the 18S rDNA gene that exhibits a low genetic diversity and can be present in several copies in *Acanthamoeba* genomes. These systems might notably be helpful to detect and solve previous incongruence in *Acanthamoeba* species and results obtained with these systems suggest that a more accurate panel of reference *Acanthamoeba* species should be delineated. In addition, these molecular systems may allow identifying new putative *Acanthamoeba* species. An accurate *Acanthamoeba* identification is needed to determine which *Acanthamoeba* species or isolate is the most efficient for the isolation by culture of giant viruses from different established or putative families. Such information will guide the choice of *Acanthamoeba* strains used as culture support to favor the isolation of additional strains of known giant viruses in order to get a better knowledge of their prevalence, diversity and pangenomes, or, alternatively, of new giant viruses.

## Supplementary information

Supplementary information.
