# Supplementary material for: Core gene-based molecular detection and identification of Acanthamoeba species
Source: Sci Rep. 2020 Jan 31;10:1583. doi: 10.1038/s41598-020-57998-5 (PMC6994504; doi:10.1038/s41598-020-57998-5)
Supplement: Supplementary file 1 — Supplementary information. [file 41598_2020_57998_MOESM1_ESM.pdf]

SUPPLEMENTARY MATERIAL TITLE PAGE

Full-length title: Core gene-based molecular detection and identification of *Acanthamoeba* species

Short title (for the running head): Molecular detection and identification of *Acanthamoeba* spp.

Author list: Nisrine CHELKHA[1,2], Priscilla JARDOT[1,2], Iness MOUSSAOUI[1,2], Anthony LEVASSEUR[1,2], Bernard LA SCOLA[1,2], Philippe COLSON[1,2*]

Affiliations: [1] Aix-Marseille Univ., Institut de Recherche pour le Développement (IRD), Assistance Publique - Hôpitaux de Marseille (AP-HM), MEPHI, 27 boulevard Jean Moulin, 13005 Marseille, France; [2] IHU Méditerranée Infection, 19-21 boulevard Jean Moulin, 13005 Marseille, France

*** Corresponding author:** Philippe Colson, IHU - Méditerranée Infection, AP-HM, 19-21 boulevard Jean Moulin, 13005 Marseille, France. Tel.: +33 413 732 401, Fax: +33 413 732 052; email: philippe.colson@univ-amu.fr

Key words: *Acanthamoeba*; identification; classification; sequencing; genotypes; giant viruses; culture isolation; human

SUPPLEMENTARY FIGURES

Figure S1. Maps of the alanine-tRNA ligase gene and the 18S rDNA gene showing where the primers anneal using the SVARAP tool.

A: Lig1_F to Lig3_R primers; B: Acant_18S_F1 to Acant_18S_R4, and the Am96F1/Ami9R primers.

Figure S1.

A

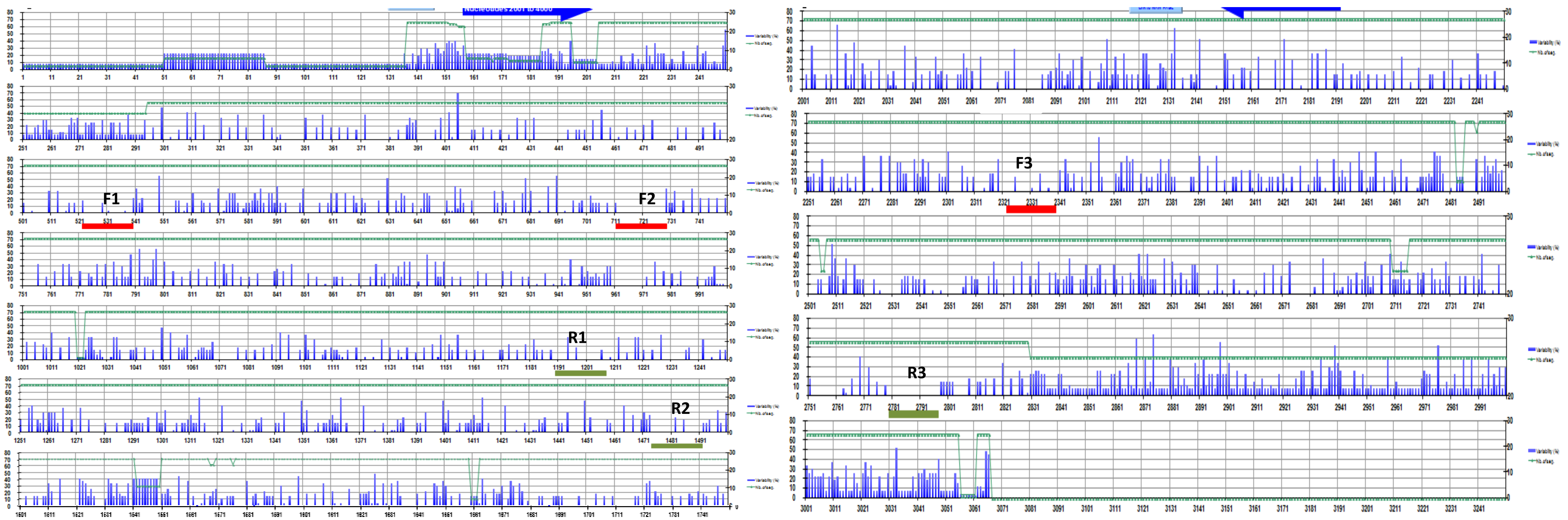

B

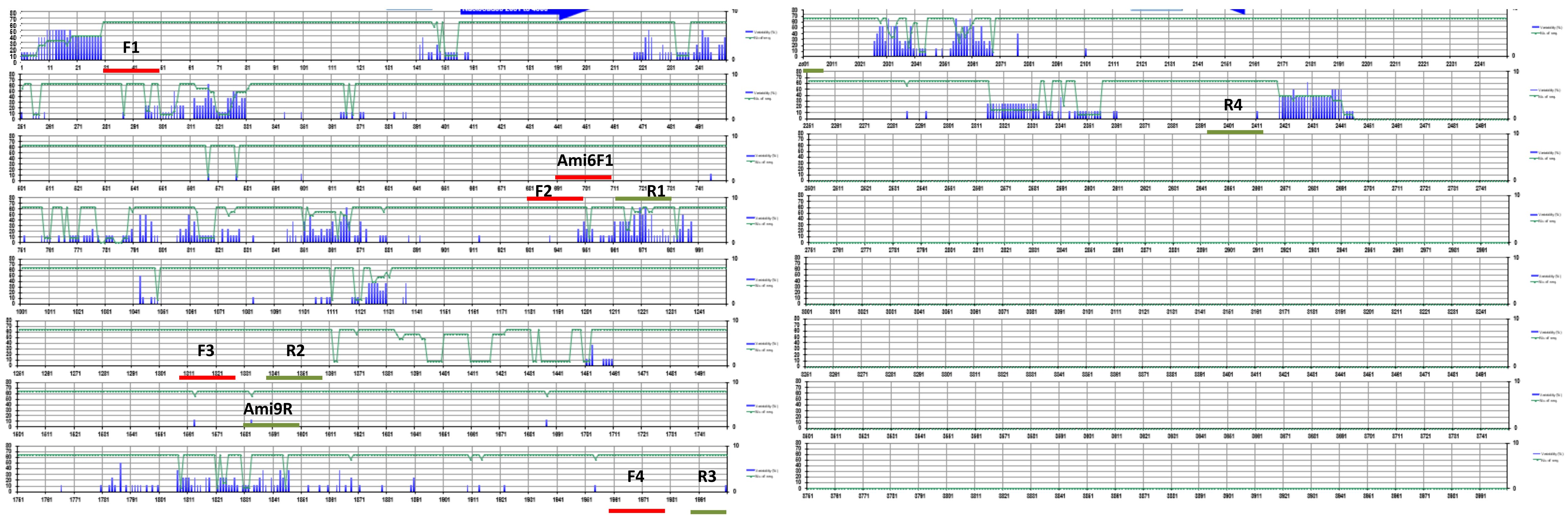

Figure S2. Identification strategy of an unknown amoeba using the alanine-tRNA ligase gene sequence.

Figure S2.

A

PCR systems	*Acanthamoeba* species detection failures	*Acanthamoeba* species identification failures
Lig1	*A. tubiashi* ATCC 30867<br>*A.* sp. clinical isolate 2<br>*A.* sp. environmental isolate 5<br>*A.* sp. environmental isolate 3	1) *A. castellanii* strain Neff, *A. palestinensis* ATCC 30870 and *A. culbertsoni* ATCC 30171<br>2) *A. astronyxis* ATCC 30137 and *A. divionensis* ATCC 50238<br>3) *A. quina* ATCC 50241, *A.* sp. clinical isolate 8 and *A.* sp. clinical isolate 7<br>4) *A. hatchetti* ATCC PRA-113 and *A.* sp. environmental isolate 1<br>5) *A.* sp. clinical isolate 4 and *A.* sp. environmental isolate 4<br>6) *A.* sp. clinical isolate 3 and *A.* sp. clinical isolate 1<br>7) *A.* sp. clinical isolate 6 and *A.* sp. environmental isolate 2
Lig2	*A. polyphaga* strain Linc-AP1<br>*A. tubiashi* ATCC 30867<br>*A.* sp. environmental isolate 5<br>*A.* sp. environmental isolate 3	1) *A. castellanii* strain Neff and *A. terricola* ATCC 30134<br>2) *A. quina* ATCC 50241 and *A.* sp. clinical isolate 6<br>3) *A. culbertsoni* ATCC 30171 and *A. lugdunensis* ATCC 50240<br>4) *A. triangularis* ATCC 50254, *A.* sp. clinical isolate 4 and *A.* sp. environmental isolate 2<br>5) *A. astronyxis* ATCC 30137 and *A. divionensis* ATCC 50238
Lig3	*A.* sp. environmental isolate 5	1) *A. polyphaga* ATCC 30872 and *A.* sp. clinical isolate 5<br>2) *A. castellanii* strain Neff, *A. triangularis* ATCC 50254 and *A.* sp. clinical isolate 1<br>3) *A. hatchetti* ATCC PRA-113 and *A.* sp. environmental isolate 1<br>4) *A. polyphaga* strain Linc-AP1 and *A. royreba* ATCC 30884<br>5) *A.* sp. clinical isolate 8, *A.* sp. clinical isolate 2, *A.* sp. environmental isolate 3, *A.* sp. environmental isolate 2, *A.* sp. clinical isolate 6 and *A.* sp. clinical isolate 7<br>6) *A.* sp. clinical isolate 4 and *A.* sp. environmental isolate 4

B

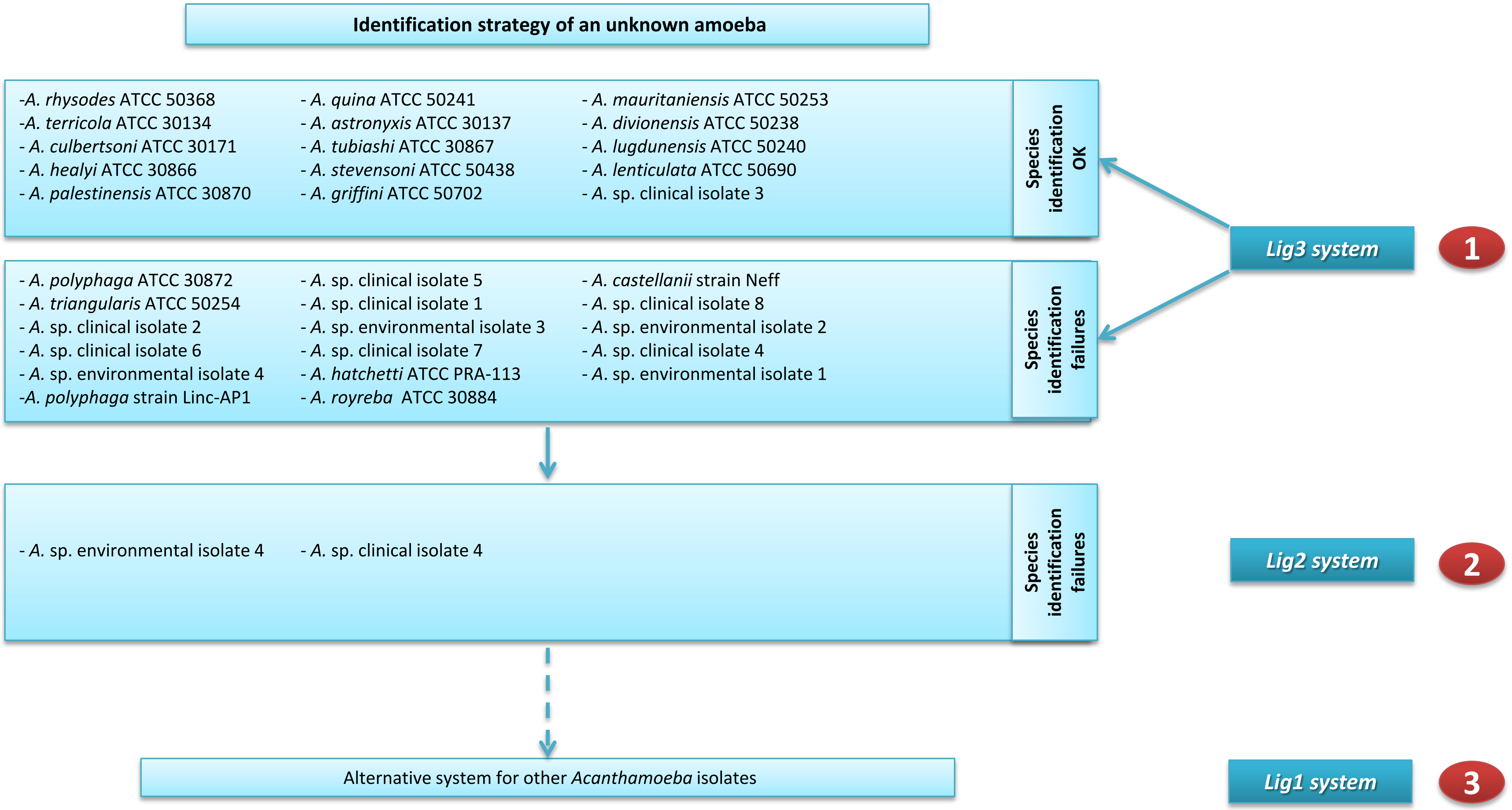

SUPPLEMENTARY TABLES

Table S1. Predicted open reading frame in the draft genome sequences of the 14 different species of *Acanthamoeba*.

Species	Draft genome sequence length (Mb)	Total number of predicted ORFs	ORFs⩾ 100 aa
Acanthamoeba castellanii	115.3	381,505	96,232
Acanthamoeba polyphaga	120.6	374,196	97,092
Acanthamoeba astronyxis	83.5	203,336	60,691
Acanthamoeba culbertsoni	55.6	129,164	46,309
Acanthamoeba divionensis	84.6	211,955	61,225
Acanthamoeba healyi	75.3	161,883	67,989
Acanthamoeba lenticulata	66.1	153,534	58,461
Acanthamoeba lugdunensis	99.5	229,323	91,448
Acanthamoeba mauritaniensis	106.9	234,464	95,443
Acanthamoeba palestinensis	103.5	203,374	90,317
Acanthamoeba pearcei	115.8	386,877	95,162
Acanthamoeba quina	83.6	195,999	75,556
Acanthamoeba rhysodes	75.9	186,569	68,073
Acanthamoeba royreba	79.6	171,918	69,549

aa, amino acids; Mb, megabase pairs; ORF, open reading frame

Table S2. Candidate genes found to have nucleotide sequences that differ in each of the 14 species.

Function	Length (aa)
Alanine-tRNA ligase	954
BAR domain containing protein	165
NAD binding domain 4 domain containing protein	288
Gpatch domain containing protein	230
Helicase conserved Cterminal domain containing protein	383
K+ channel tetramerisation subfamily protein	197
Lalate/lactate dehydrogenase	443
Mitogen-activated protein kinase kinase	151
NAD(P)H:quinone oxidoreductase	160
PB1 domain containing protein	346
Peptidase M16 family protein	377
DNA dependent RNA polymerase	419
Surp module domain containing protein	597
THUMP domain containing 3 isoform 1_putative	295
Ubiquitin carboxyl-terminal hydrolase	548

aa, amino acids

Table S3. Sequence similarities between PCR primers and targeted regions, and amplicons identity between *Acanthamoeba* species.

	Nucleotide identity with *Acanthamoeba* genomes (%)	Nucleotide identity between amplicons (%)
Lig1_F	94[1]-100	67-100
Lig1_R	94[2]-100	
Lig2_F	100	45-100
Lig2_R	89[3]-100	
Lig3_F	93[4]-100	55-100
Lig3_R	100	

[1] For *A. culbertsoni*, *A. astronyxis* and *A. divionensis*.
[2] For *A. culbertsoni*, *A. astronyxis, A. divionensis* and *A. lenticulata*.
[3] For *A. astronyxis* and *A. divionensis*.
[4] For *A. culbertsoni*, *A. astronyxis* and *A. divionensis*.

Tables S4. Identification accuracy of *Acanthamoeba* species based on a nucleotide similarity <100% and a bootstrap value <90%, a pairwise similarity matrices between sequences from reference strains and environmental and clinical isolates for each PCR system (a), and nucleotide sequence identity matrices for *Acanthamoeba* 18S rDNA gene fragments obtained with PCR primers Ami6F1 and Ami9R (b), for sequences from complete *Acanthamoeba* 18S rDNA gene (c), for *Acanthamoeba* alanine-tRNA ligase gene fragments obtained with PCR primer systems Lig1 (d), Lig2 (e) and Lig3 (f), and for concatenated *Acanthamoeba* alanine-tRNA ligase gene fragments obtained with PCR primer systems Lig1, Lig2 and Lig3 (g).

Table S4a.

		Identification using only Bootstrap <90%					
		18S Ami6F1/9R	18S complete	Lig1	Lig2	Lig3	Lig concat
Reference strains	Acanthamoeba rhysodes ATCC 50368	13	17	13	13	8	15
	Acanthamoeba polyphaga ATCC 30872	3	3	1	11	2	1
	Acanthamoeba castellanii strain Neff	16	11	14	16	7	4
	Acanthamoeba polyphaga strain Linc-AP1	1	1	17	-	3	-
	Acanthamoeba royreba ATCC 30884	5	5	24	25	3	20
	Acanthamoeba healyi ATCC 30866	8	8	20	23	25	19
	Acanthamoeba palestinensis ATCC 30870	8	8	16	15	9	4
	Acanthamoeba lenticulata ATCC 50690	21	20	23	24	27	21
	Acanthamoeba quina ATCC 50241	15	12	9	9	14	12
	Acanthamoeba terricola ATCC 30134	4	4	18	17	6	4
	Acanthamoeba culbertsoni ATCC 30171	22	21	15	1	28	16
	Acanthamoeba stevensoni ATCC 50438	20	19	22	21	26	18
	Acanthamoeba griffini ATCC 50702	7	10	21	22	15	17
	Acanthamoeba lugdunensis ATCC 50240	1	1	12	1	24	6
	Acanthamoeba mauritaniensis ATCC 50253	5	5	19	20	4	14
	Acanthamoeba hatchetti ATCC PRA-113	18	7	2	18	1	2
	Acanthamoeba triangularis ATCC 50254	17	18	3	2	12	3
	Acanthamoeba astronyxis ATCC 30137	9	9	4	3	29	5
	Acanthamoeba divionensis ATCC 50238	5	5	4	3	5	5
	Acanthamoeba tubiashi ATCC 30867	9	9	-	-	16	-
isolates	Acanthamoeba sp. clinical isolate 1	4	4	6	14	13	13
	Acanthamoeba sp. clinical isolate 2	1	1	-	5	19	-
	Acanthamoeba sp. clinical isolate 3	6	6	5	6	17	9
	Acanthamoeba sp. clinical isolate 4	6	6	3	2	10	3
	Acanthamoeba sp. clinical isolate 5	11	13	1	12	2	1
	Acanthamoeba sp. clinical isolate 6	14	16	11	8	22	8
	Acanthamoeba sp. clinical isolate 7	2	2	7	4	23	11
	Acanthamoeba sp. clinical isolate 8	2	2	8	7	18	10
	Acanthamoeba sp. environmental isolate 1	19	7	2	19	1	2
	Acanthamoeba sp. environmental isolate 2	10	15	10	10	21	7
	Acanthamoeba sp. environmental isolate 3	12	14	-	-	20	-
	Acanthamoeba sp. environmental isolate 4	6	6	3	2	11	3
	Acanthamoeba sp. environmental isolate 5	3	3	-	-	-	-

		Identification using only Similarity <100%					
		18S Ami6F1/9R	18S complete	Lig1	Lig2	Lig3	Lig concat
Reference strains	Acanthamoeba rhysodes ATCC 50368	5	1	6	4	5	1
	Acanthamoeba polyphaga ATCC 30872	6	2	7	5	1	2
	Acanthamoeba castellanii strain Neff	7	3	1	1	2	3
	Acanthamoeba polyphaga strain Linc-AP1	8	4	8	-	6	-
	Acanthamoeba royreba ATCC 30884	9	5	9	6	7	4
	Acanthamoeba healyi ATCC 30866	4	6	10	7	8	5
	Acanthamoeba palestinensis ATCC 30870	4	7	1	8	9	6
	Acanthamoeba lenticulata ATCC 50690	10	8	11	9	10	7
	Acanthamoeba quina ATCC 50241	11	9	2	2	11	8
	Acanthamoeba terricola ATCC 30134	12	10	12	1	12	9
	Acanthamoeba culbertsoni ATCC 30171	13	11	1	3	13	10
	Acanthamoeba stevensoni ATCC 50438	14	12	13	10	14	11
	Acanthamoeba griffini ATCC 50702	15	13	14	11	15	12
	Acanthamoeba lugdunensis ATCC 50240	1	14	15	3	16	13
	Acanthamoeba mauritaniensis ATCC 50253	16	15	16	12	17	14
	Acanthamoeba hatchetti ATCC PRA-113	17	16	17	13	18	15
	Acanthamoeba triangularis ATCC 50254	18	17	18	14	2	16
	Acanthamoeba astronyxis ATCC 30137	19	18	19	15	19	17
	Acanthamoeba divionensis ATCC 50238	20	19	20	16	20	18
	Acanthamoeba tubiashi ATCC 30867	21	20	-	-	21	-
isolates	Acanthamoeba sp. clinical isolate 1	27	32	4	26	2	29
	Acanthamoeba sp. clinical isolate 2	1	23	-	19	3	21
	Acanthamoeba sp. clinical isolate 3	2	21	4	17	22	19
	Acanthamoeba sp. clinical isolate 4	2	24	3	20	4	22
	Acanthamoeba sp. clinical isolate 5	23	26	22	22	1	24
	Acanthamoeba sp. clinical isolate 6	26	29	5	2	3	26
	Acanthamoeba sp. clinical isolate 7	3	30	2	24	3	27
	Acanthamoeba sp. clinical isolate 8	3	22	2	18	3	20
	Acanthamoeba sp. environmental isolate 1	22	25	21	21	23	23
	Acanthamoeba sp. environmental isolate 2	25	28	5	23	3	25
	Acanthamoeba sp. environmental isolate 3	24	27	-	-	3	-
	Acanthamoeba sp. environmental isolate 4	2	31	3	25	4	28
	Acanthamoeba sp. environmental isolate 5	28	33	-	-	-	-

- Not detected using PCR and/or sequencing

		Identification using both Bootstrap <90% and Similarity <100%					
		18S Ami6F1/9R	18S complete	Lig1	Lig2	Lig3	Lig concat
Reference strains	Acanthamoeba rhysodes ATCC 50368	Yes	Yes	Yes	Yes	Yes	Yes
	Acanthamoeba polyphaga ATCC 30872	Yes	Yes	Yes	Yes	Yes	Yes
	Acanthamoeba castellanii strain Neff	Yes	Yes	No	No	No	No
	Acanthamoeba polyphaga strain Linc-AP1	No	No	Yes	-	No	-
	Acanthamoeba royreba ATCC 30884	No	No	Yes	Yes	No	Yes
	Acanthamoeba healyi ATCC 30866	No	No	Yes	Yes	Yes	Yes
	Acanthamoeba palestinensis ATCC 30870	No	No	No	Yes	Yes	No
	Acanthamoeba lenticulata ATCC 50690	Yes	Yes	Yes	Yes	Yes	Yes
	Acanthamoeba quina ATCC 50241	Yes	Yes	Yes	Yes	Yes	Yes
	Acanthamoeba terricola ATCC 30134	Yes	Yes	Yes	No	Yes	No
	Acanthamoeba culbertsoni ATCC 30171	Yes	Yes	No	No	Yes	Yes
	Acanthamoeba stevensoni ATCC 50438	Yes	Yes	Yes	Yes	Yes	Yes
	Acanthamoeba griffini ATCC 50702	Yes	Yes	Yes	Yes	Yes	Yes
	Acanthamoeba lugdunensis ATCC 50240	No	No	Yes	No	Yes	Yes
	Acanthamoeba mauritaniensis ATCC 50253	No	No	Yes	Yes	Yes	Yes
	Acanthamoeba hatchetti ATCC PRA-113	Yes	Yes	Yes	Yes	Yes	Yes
	Acanthamoeba triangularis ATCC 50254	Yes	Yes	Yes	Yes	No	Yes
	Acanthamoeba astronyxis ATCC 30137	No	No	No	No	Yes	No
	Acanthamoeba divionensis ATCC 50238	No	No	No	No	Yes	No
	Acanthamoeba tubiashi ATCC 30867	No	No	-	-	Yes	-
isolates	Acanthamoeba sp. clinical isolate 1	Yes	Yes	No	No	Yes	No
	Acanthamoeba sp. clinical isolate 2	Yes	Yes	-	No	No	-
	Acanthamoeba sp. clinical isolate 3	No	No	No	No	No	No
	Acanthamoeba sp. clinical isolate 4	No	No	Yes	Yes	No	Yes
	Acanthamoeba sp. clinical isolate 5	No	No	Yes	No	Yes	Yes
	Acanthamoeba sp. clinical isolate 6	No	No	No	Yes	No	No
	Acanthamoeba sp. clinical isolate 7	No	No	Yes	No	No	No
	Acanthamoeba sp. clinical isolate 8	No	No	Yes	No	No	No
	Acanthamoeba sp. environmental isolate 1	No	Yes	Yes	No	Yes	Yes
	Acanthamoeba sp. environmental isolate 2	No	No	No	No	No	No
	Acanthamoeba sp. environmental isolate 3	No	No	-	-	No	-
	Acanthamoeba sp. environmental isolate 4	No	No	Yes	Yes	No	Yes
	Acanthamoeba sp. environmental isolate 5	Yes	Yes	-	-	-	-
	**Identification ofreference strains**	11/20	11/20	14/19	12/18	16/20	13/18
	**Identification of isolates based on reference strains**	3/13	4/13	6/10	3/11	3/12	4/10

Table S4b.

TabS4_Chelkha_Colson_SciRep_Apr2019_vL 18S ami6F&9R

Seq->	18S A. lugdunensis	18S A. stevensoni	18S A. griffini	Acanthamoeba sp. clinical isolate 6	Acanthamoeba sp. environmental isolate 4	18S A. hatchetti	Acanthamoeba sp. environmental isolate 1	Acanthamoeba sp. environmental isolate 2	Acanthamoeba sp. clinical isolate 4	Acanthamoeba sp. clinical isolate 3	18S A. terricola	Acanthamoeba sp. clinical isolate 1	Acanthamoeba sp. clinical isolate 5	Acanthamoeba sp. environmental isolate 5	Acanthamoeba sp. environmental isolate 3	Acanthamoeba sp. clinical isolate 8	Acanthamoeba sp. clinical isolate 7	Acanthamoeba sp. clinical isolate 2	18S A, polyphaga Linc-AP1	EF554328.1 Acanthamoeba castellanii strain Neff ATCC 30010 18S ribosomal RNA gene partial sequence	AF019065.1 Acanthamoeba tubiashi OC15C 18S ribosomal RNA gene partial sequence	AF019064.1 Acanthamoeba astronyxis Ray Hayes 18S ribosomal RNA gene partial sequence	AF019067.1 Acanthamoeba culbertsoni Lilly A1 18S ribosomal RNA gene partial sequence	U94735.1 Acanthamoeba lenticulata strain E182 18S small subunit ribosomal RNA gene partial sequence	AY026244.1 Acanthamoeba polyphaga strain ATCC30872 small subunit ribosomal RNA gene complete sequence	U07411.1 Acanthamoeba palestinensis Reich ATCC 30870 18S rRNA gene	AF019053.1 Acanthamoeba pearcei 18S ribosomal RNA gene partial sequence	AF346662.1 Acanthamoeba triangularis 18S ribosomal RNA gene partial sequence	AY351646.1 Acanthamoeba divionensis strain AA2 18S ribosomal RNA gene complete sequence	U07417.1 Acanthamoeba royreba Oak Rge ATCC 30884 18S rRNA gene	AY351647.1 Acanthamoeba mauritaniensis 18S ribosomal RNA gene complete sequence	U07414.1 Acanthamoeba castellanii Ma ATCC 50370 18S rRNA gene	U07406.1 Acanthamoeba rhysodes BCM:0685:116 ATCC 50368 18S rRNA gene	18S A. healyi	18S A. quina	18S A, polyphaga ATCC 30872
18S A. lugdunensis		0,917	0,928	0,912	0,971	0,946	0,946	0,958	0,971	0,971	0,955	0,956	0,972	0,954	0,972	0,983	0,983	1,000	0,998	0,991	0,669	0,609	0,807	0,451	0,846	0,859	0,918	0,960	0,942	0,955	0,956	0,960	0,982	0,859	0,986	0,878
18S A. stevensoni	0,917		0,901	0,852	0,922	0,913	0,908	0,903	0,922	0,922	0,906	0,908	0,908	0,897	0,915	0,907	0,907	0,917	0,916	0,914	0,647	0,587	0,812	0,440	0,816	0,839	0,901	0,897	0,906	0,912	0,911	0,917	0,919	0,839	0,915	0,834
18S A. griffini	0,928	0,901		0,853	0,926	0,918	0,920	0,904	0,926	0,926	0,917	0,917	0,918	0,918	0,917	0,920	0,920	0,928	0,926	0,927	0,648	0,597	0,809	0,443	0,847	0,858	0,956	0,913	0,892	0,905	0,904	0,935	0,922	0,858	0,924	0,843
Acanthamoeba sp. clinical isolate 6	0,912	0,852	0,853		0,892	0,880	0,877	0,885	0,892	0,892	0,890	0,890	0,894	0,882	0,914	0,906	0,906	0,912	0,911	0,909	0,622	0,568	0,744	0,423	0,770	0,794	0,842	0,876	0,874	0,884	0,885	0,886	0,907	0,794	0,918	0,812
Acanthamoeba sp. environmental isolate 4	0,971	0,922	0,926	0,892		0,950	0,948	0,939	1,000	1,000	0,943	0,944	0,965	0,939	0,960	0,960	0,960	0,971	0,970	0,967	0,662	0,609	0,801	0,449	0,850	0,860	0,919	0,958	0,941	0,950	0,951	0,955	0,967	0,860	0,962	0,872
18S A. hatchetti	0,946	0,913	0,918	0,880	0,950		0,982	0,917	0,950	0,950	0,941	0,941	0,945	0,934	0,938	0,934	0,934	0,946	0,944	0,945	0,660	0,602	0,794	0,446	0,838	0,854	0,900	0,933	0,916	0,924	0,925	0,944	0,942	0,854	0,943	0,853
Acanthamoeba sp. environmental isolate 1	0,946	0,908	0,920	0,877	0,948	0,982		0,915	0,948	0,948	0,946	0,946	0,942	0,938	0,935	0,941	0,941	0,946	0,944	0,949	0,658	0,600	0,795	0,445	0,842	0,856	0,902	0,937	0,913	0,931	0,933	0,949	0,940	0,856	0,951	0,861
Acanthamoeba sp. environmental isolate 2	0,958	0,903	0,904	0,885	0,939	0,917	0,915		0,939	0,939	0,941	0,942	0,948	0,921	0,951	0,950	0,950	0,958	0,956	0,956	0,659	0,608	0,788	0,443	0,824	0,840	0,900	0,928	0,934	0,945	0,947	0,926	0,956	0,840	0,949	0,849
Acanthamoeba sp. clinical isolate 4	0,971	0,922	0,926	0,892	1,000	0,950	0,948	0,939		1,000	0,943	0,944	0,965	0,939	0,960	0,960	0,960	0,971	0,970	0,967	0,662	0,609	0,801	0,449	0,850	0,860	0,919	0,958	0,941	0,950	0,951	0,955	0,967	0,860	0,962	0,872
Acanthamoeba sp. clinical isolate 3	0,971	0,922	0,926	0,892	1,000	0,950	0,948	0,939	1,000		0,943	0,944	0,965	0,939	0,960	0,960	0,960	0,971	0,970	0,967	0,662	0,609	0,801	0,449	0,850	0,860	0,919	0,958	0,941	0,950	0,951	0,955	0,967	0,860	0,962	0,872
18S A. terricola	0,955	0,906	0,917	0,890	0,943	0,941	0,946	0,941	0,943	0,943		0,997	0,946	0,934	0,947	0,945	0,945	0,955	0,954	0,961	0,659	0,600	0,805	0,447	0,832	0,850	0,908	0,936	0,922	0,941	0,942	0,942	0,947	0,850	0,964	0,863
Acanthamoeba sp. clinical isolate 1	0,956	0,908	0,917	0,890	0,944	0,941	0,946	0,942	0,944	0,944	0,997		0,947	0,934	0,947	0,946	0,946	0,956	0,955	0,963	0,660	0,601	0,806	0,447	0,833	0,851	0,909	0,937	0,924	0,942	0,943	0,942	0,948	0,851	0,964	0,863
Acanthamoeba sp. clinical isolate 5	0,972	0,908	0,918	0,894	0,965	0,945	0,942	0,948	0,965	0,965	0,946	0,947		0,944	0,958	0,961	0,961	0,972	0,971	0,971	0,668	0,612	0,796	0,451	0,844	0,856	0,909	0,951	0,933	0,942	0,943	0,951	0,967	0,856	0,964	0,862
Acanthamoeba sp. environmental isolate 5	0,954	0,897	0,918	0,882	0,939	0,934	0,938	0,921	0,939	0,939	0,934	0,934	0,944		0,947	0,947	0,947	0,954	0,953	0,950	0,655	0,593	0,791	0,449	0,839	0,858	0,910	0,935	0,909	0,927	0,929	0,969	0,943	0,858	0,955	0,877
Acanthamoeba sp. environmental isolate 3	0,972	0,915	0,917	0,914	0,960	0,938	0,935	0,951	0,960	0,960	0,947	0,947	0,958	0,947		0,971	0,971	0,972	0,971	0,966	0,668	0,610	0,793	0,446	0,834	0,854	0,909	0,940	0,941	0,949	0,950	0,949	0,982	0,854	0,981	0,874
Acanthamoeba sp. clinical isolate 8	0,983	0,907	0,920	0,906	0,960	0,934	0,941	0,950	0,960	0,960	0,945	0,946	0,961	0,947	0,971		1,000	0,983	0,982	0,980	0,667	0,606	0,796	0,447	0,838	0,856	0,910	0,949	0,936	0,954	0,955	0,951	0,978	0,856	0,977	0,874
Acanthamoeba sp. clinical isolate 7	0,983	0,907	0,920	0,906	0,960	0,934	0,941	0,950	0,960	0,960	0,945	0,946	0,961	0,947	0,971	1,000		0,983	0,982	0,980	0,667	0,606	0,796	0,447	0,838	0,856	0,910	0,949	0,936	0,954	0,955	0,951	0,978	0,856	0,977	0,874
Acanthamoeba sp. clinical isolate 2	1,000	0,917	0,928	0,912	0,971	0,946	0,946	0,958	0,971	0,971	0,955	0,956	0,972	0,954	0,972	0,983	0,983		0,998	0,991	0,669	0,609	0,807	0,451	0,846	0,859	0,918	0,960	0,942	0,955	0,956	0,960	0,982	0,859	0,986	0,878
18S A, polyphaga Linc-AP1	0,998	0,916	0,926	0,911	0,970	0,944	0,944	0,956	0,970	0,970	0,954	0,955	0,971	0,953	0,971	0,982	0,982	0,998		0,990	0,668	0,608	0,806	0,450	0,845	0,858	0,917	0,958	0,940	0,954	0,955	0,959	0,981	0,858	0,985	0,877
EF554328.1 Acanthamoeba castellanii strain Neff ATCC	0,991	0,914	0,927	0,909	0,967	0,945	0,949	0,956	0,967	0,967	0,961	0,963	0,971	0,950	0,966	0,980	0,980	0,991	0,990		0,668	0,609	0,805	0,450	0,846	0,858	0,917	0,958	0,939	0,957	0,959	0,959	0,978	0,858	0,985	0,878
AF019065.1 Acanthamoeba tubiashi OC15C 18S ribosor	0,669	0,647	0,648	0,622	0,662	0,660	0,658	0,659	0,662	0,662	0,659	0,660	0,668	0,655	0,668	0,667	0,667	0,669	0,668	0,668		0,743	0,619	0,371	0,624	0,632	0,645	0,656	0,655	0,662	0,662	0,653	0,671	0,632	0,666	0,601
AF019064.1 Acanthamoeba astronyxis Ray Hayes 18S r	0,609	0,587	0,597	0,568	0,609	0,602	0,600	0,608	0,609	0,609	0,600	0,601	0,612	0,593	0,610	0,606	0,606	0,609	0,608	0,609	0,743		0,561	0,357	0,581	0,578	0,593	0,598	0,590	0,596	0,596	0,594	0,609	0,578	0,609	0,552
AF019067.1 Acanthamoeba culbertsoni Lilly A1 18S ribc	0,807	0,812	0,809	0,744	0,801	0,794	0,795	0,788	0,801	0,801	0,805	0,806	0,796	0,791	0,793	0,796	0,796	0,807	0,806	0,805	0,619	0,561		0,410	0,777	0,775	0,803	0,795	0,790	0,800	0,799	0,808	0,801	0,775	0,800	0,741
U94735.1 Acanthamoeba lenticulata strain E182 18S sm	0,451	0,440	0,443	0,423	0,449	0,446	0,445	0,443	0,449	0,449	0,447	0,447	0,451	0,449	0,446	0,447	0,447	0,451	0,450	0,450	0,371	0,357	0,410		0,443	0,446	0,444	0,443	0,442	0,447	0,447	0,449	0,449	0,446	0,448	0,414
AY026244.1 Acanthamoeba polyphaga strain ATCC3087	0,846	0,816	0,847	0,770	0,850	0,838	0,842	0,824	0,850	0,850	0,832	0,833	0,844	0,839	0,834	0,838	0,838	0,846	0,845	0,846	0,624	0,581	0,777	0,443		0,888	0,841	0,841	0,827	0,840	0,840	0,848	0,844	0,888	0,841	0,772
U07411.1 Acanthamoeba palestinensis Reich ATCC 308	0,859	0,839	0,858	0,794	0,860	0,854	0,856	0,840	0,860	0,860	0,850	0,851	0,856	0,858	0,854	0,856	0,856	0,859	0,858	0,858	0,632	0,578	0,775	0,446	0,888		0,852	0,854	0,848	0,861	0,859	0,869	0,856	1,000	0,859	0,792
AF019053.1 Acanthamoeba pearcei 18S ribosomal RNA	0,918	0,901	0,956	0,842	0,919	0,900	0,902	0,900	0,919	0,919	0,908	0,909	0,909	0,910	0,909	0,910	0,910	0,918	0,917	0,917	0,645	0,593	0,803	0,444	0,841	0,852		0,910	0,893	0,906	0,905	0,927	0,912	0,852	0,914	0,839
AF346662.1 Acanthamoeba triangularis 18S ribosomal F	0,960	0,897	0,913	0,876	0,958	0,933	0,937	0,928	0,958	0,958	0,936	0,937	0,951	0,935	0,940	0,949	0,949	0,960	0,958	0,958	0,656	0,598	0,795	0,443	0,841	0,854	0,910		0,916	0,930	0,931	0,945	0,950	0,854	0,954	0,860
AY351646.1 Acanthamoeba divionensis strain AA2 18S r	0,942	0,906	0,892	0,874	0,941	0,916	0,913	0,934	0,941	0,941	0,922	0,924	0,933	0,909	0,941	0,936	0,936	0,942	0,940	0,939	0,655	0,590	0,790	0,442	0,827	0,848	0,893	0,916		0,981	0,980	0,916	0,949	0,848	0,938	0,841
U07417.1 Acanthamoeba royreba Oak Rge ATCC 30884	0,955	0,912	0,905	0,884	0,950	0,924	0,931	0,945	0,950	0,950	0,941	0,942	0,942	0,927	0,949	0,954	0,954	0,955	0,954	0,957	0,662	0,596	0,800	0,447	0,840	0,861	0,906	0,930	0,981		0,998	0,934	0,957	0,861	0,956	0,859
AY351647.1 Acanthamoeba mauritaniensis 18S ribosom	0,956	0,911	0,904	0,885	0,951	0,925	0,933	0,947	0,951	0,951	0,942	0,943	0,943	0,929	0,950	0,955	0,955	0,956	0,955	0,959	0,662	0,596	0,799	0,447	0,840	0,859	0,905	0,931	0,980	0,998		0,935	0,959	0,859	0,958	0,860
U07414.1 Acanthamoeba castellanii Ma ATCC 50370 18	0,960	0,917	0,935	0,886	0,955	0,944	0,949	0,926	0,955	0,955	0,942	0,942	0,951	0,969	0,949	0,951	0,951	0,960	0,959	0,959	0,653	0,594	0,808	0,449	0,848	0,869	0,927	0,945	0,916	0,934	0,935		0,949	0,869	0,961	0,893
U07406.1 Acanthamoeba rhysodes BCM:0685:116 ATC(	0,982	0,919	0,922	0,907	0,967	0,942	0,940	0,956	0,967	0,967	0,947	0,948	0,967	0,943	0,982	0,978	0,978	0,982	0,981	0,978	0,671	0,609	0,801	0,449	0,844	0,856	0,912	0,950	0,949	0,957	0,959	0,949		0,856	0,974	0,871
18S A. healyi	0,859	0,839	0,858	0,794	0,860	0,854	0,856	0,840	0,860	0,860	0,850	0,851	0,856	0,858	0,854	0,856	0,856	0,859	0,858	0,858	0,632	0,578	0,775	0,446	0,888	1,000	0,852	0,854	0,848	0,861	0,859	0,869	0,856		0,859	0,792
18S A. quina	0,986	0,915	0,924	0,918	0,962	0,943	0,951	0,949	0,962	0,962	0,964	0,964	0,964	0,955	0,981	0,977	0,977	0,986	0,985	0,985	0,666	0,609	0,800	0,448	0,841	0,859	0,914	0,954	0,938	0,956	0,958	0,961	0,974	0,859		0,883
18S A, polyphaga ATCC 30872	0,878	0,834	0,843	0,812	0,872	0,853	0,861	0,849	0,872	0,872	0,863	0,863	0,862	0,877	0,874	0,874	0,874	0,878	0,877	0,878	0,601	0,552	0,741	0,414	0,772	0,792	0,839	0,860	0,841	0,859	0,860	0,893	0,871	0,792	0,883	

Table S4c.

Seq->	18S A. lugdunensis	18S A. stevensoni	18S A. griffini	Acanthamoeba sp. clinical isolate 6	Acanthamoeba sp. environmental isolate 4	18S A. hatchetti	Acanthamoeba sp. environmental isolate 1	Acanthamoeba sp. environmental isolate 2	Acanthamoeba sp. clinical isolate 4	Acanthamoeba sp. clinical isolate 3	18S A. terricola	Acanthamoeba sp. clinical isolate 1	Acanthamoeba sp. clinical isolate 5	Acanthamoeba sp. environmental isolate 5	Acanthamoeba sp. environmental isolate 3	Acanthamoeba sp. clinical isolate 8	Acanthamoeba sp. clinical isolate 7	Acanthamoeba sp. clinical isolate 2	18S A, polyphaga Linc-AP1	EF554328.1 Acanthamoeba castellanii strain Neff ATCC 30010 18S ribosomal RNA gene partial sequence	AF019065.1 Acanthamoeba tubiashi OC15C 18S ribosomal RNA gene partial sequence	AF019064.1 Acanthamoeba astronyxis Ray Hayes 18S ribosomal RNA gene partial sequence	AF019067.1 Acanthamoeba culbertsoni Lilly A1 18S ribosomal RNA gene partial sequence	U94735.1 Acanthamoeba lenticulata strain E182 18S small subunit ribosomal RNA gene partial sequence	AY026244.1 Acanthamoeba polyphaga strain ATCC30872 small subunit ribosomal RNA gene complete sequence	U07411.1 Acanthamoeba palestinensis Reich ATCC 30870 18S rRNA gene	AF019053.1 Acanthamoeba pearcei 18S ribosomal RNA gene partial sequence	AF346662.1 Acanthamoeba triangularis 18S ribosomal RNA gene partial sequence	AY351646.1 Acanthamoeba divionensis strain AA2 18S ribosomal RNA gene complete sequence	U07417.1 Acanthamoeba royreba Oak Rge ATCC 30884 18S rRNA gene	AY351647.1 Acanthamoeba mauritaniensis 18S ribosomal RNA gene complete sequence	U07414.1 Acanthamoeba castellanii Ma ATCC 50370 18S rRNA gene	U07406.1 Acanthamoeba rhysodes BCM:0685:116 ATCC 50368 18S rRNA gene	18S A. healyi	18S A. quina	18S A, polyphaga ATCC 30872
18S A. lugdunensis		0,938	0,774	0,860	0,958	0,964	0,963	0,968	0,968	0,967	0,971	0,972	0,984	0,974	0,980	0,992	0,992	0,997	0,998	0,994	0,728	0,699	0,845	0,648	0,881	0,896	0,932	0,964	0,962	0,970	0,955	0,978	0,988	0,732	0,989	0,943
18S A. stevensoni	0,938		0,769	0,820	0,927	0,934	0,932	0,923	0,936	0,935	0,932	0,933	0,934	0,933	0,931	0,934	0,934	0,938	0,937	0,936	0,710	0,686	0,840	0,643	0,868	0,881	0,931	0,925	0,928	0,934	0,919	0,941	0,940	0,719	0,936	0,909
18S A. griffini	0,774	0,769		0,751	0,764	0,773	0,774	0,757	0,771	0,771	0,772	0,772	0,772	0,774	0,768	0,773	0,773	0,773	0,773	0,773	0,605	0,587	0,706	0,554	0,734	0,742	0,809	0,762	0,764	0,768	0,757	0,779	0,774	0,762	0,774	0,751
Acanthamoeba sp. clinical isolate 6	0,860	0,820	0,751		0,830	0,842	0,840	0,846	0,839	0,839	0,847	0,847	0,853	0,849	0,858	0,857	0,858	0,860	0,860	0,861	0,644	0,620	0,739	0,581	0,769	0,781	0,811	0,835	0,841	0,848	0,835	0,850	0,862	0,692	0,863	0,823
Acanthamoeba sp. environmental isolate 4	0,958	0,927	0,764	0,830		0,950	0,948	0,935	0,983	0,982	0,951	0,952	0,956	0,946	0,950	0,953	0,952	0,955	0,956	0,956	0,717	0,689	0,835	0,636	0,869	0,882	0,920	0,950	0,944	0,949	0,936	0,952	0,955	0,721	0,953	0,920
18S A. hatchetti	0,964	0,934	0,773	0,842	0,950		0,993	0,945	0,958	0,957	0,962	0,963	0,956	0,953	0,960	0,961	0,960	0,963	0,963	0,962	0,721	0,689	0,839	0,644	0,882	0,897	0,930	0,951	0,949	0,958	0,943	0,958	0,965	0,734	0,964	0,930
Acanthamoeba sp. environmental isolate 1	0,963	0,932	0,774	0,840	0,948	0,993		0,943	0,957	0,956	0,963	0,964	0,954	0,954	0,958	0,963	0,962	0,963	0,962	0,963	0,720	0,688	0,841	0,643	0,883	0,898	0,931	0,952	0,948	0,960	0,946	0,960	0,963	0,735	0,966	0,933
Acanthamoeba sp. environmental isolate 2	0,968	0,923	0,757	0,846	0,935	0,945	0,943		0,944	0,943	0,953	0,954	0,961	0,950	0,962	0,965	0,964	0,968	0,968	0,967	0,714	0,685	0,828	0,635	0,868	0,880	0,914	0,938	0,946	0,953	0,939	0,954	0,967	0,719	0,967	0,921
Acanthamoeba sp. clinical isolate 4	0,968	0,936	0,771	0,839	0,983	0,958	0,957	0,944		0,999	0,960	0,961	0,967	0,958	0,956	0,964	0,964	0,966	0,967	0,966	0,726	0,695	0,841	0,644	0,878	0,893	0,929	0,960	0,956	0,961	0,948	0,962	0,966	0,729	0,962	0,930
Acanthamoeba sp. clinical isolate 3	0,967	0,935	0,771	0,839	0,982	0,957	0,956	0,943	0,999		0,959	0,960	0,966	0,957	0,956	0,963	0,963	0,965	0,966	0,965	0,726	0,695	0,841	0,643	0,877	0,892	0,928	0,959	0,955	0,960	0,947	0,962	0,965	0,729	0,961	0,929
18S A. terricola	0,971	0,932	0,772	0,847	0,951	0,962	0,963	0,953	0,960	0,959		0,998	0,964	0,960	0,963	0,966	0,966	0,970	0,970	0,973	0,723	0,693	0,841	0,641	0,876	0,891	0,927	0,948	0,948	0,958	0,944	0,965	0,965	0,729	0,974	0,933
Acanthamoeba sp. clinical isolate 1	0,972	0,933	0,772	0,847	0,952	0,963	0,964	0,954	0,961	0,960	0,998		0,965	0,960	0,964	0,967	0,967	0,971	0,971	0,973	0,725	0,694	0,842	0,642	0,877	0,893	0,928	0,949	0,948	0,959	0,944	0,965	0,966	0,729	0,974	0,933
Acanthamoeba sp. clinical isolate 5	0,984	0,934	0,772	0,853	0,956	0,956	0,954	0,961	0,967	0,966	0,964	0,965		0,971	0,972	0,980	0,980	0,983	0,983	0,982	0,729	0,700	0,842	0,647	0,875	0,894	0,926	0,957	0,962	0,965	0,952	0,970	0,979	0,730	0,978	0,934
Acanthamoeba sp. environmental isolate 5	0,974	0,933	0,774	0,849	0,946	0,953	0,954	0,950	0,958	0,957	0,960	0,960	0,971		0,965	0,974	0,973	0,973	0,972	0,971	0,725	0,693	0,841	0,645	0,878	0,894	0,930	0,953	0,950	0,958	0,946	0,982	0,971	0,731	0,973	0,943
Acanthamoeba sp. environmental isolate 3	0,980	0,931	0,768	0,858	0,950	0,960	0,958	0,962	0,956	0,956	0,963	0,964	0,972	0,965		0,980	0,980	0,980	0,981	0,980	0,722	0,693	0,837	0,641	0,875	0,890	0,925	0,950	0,955	0,960	0,946	0,968	0,980	0,726	0,986	0,938
Acanthamoeba sp. clinical isolate 8	0,992	0,934	0,773	0,857	0,953	0,961	0,963	0,965	0,964	0,963	0,966	0,967	0,980	0,974	0,980		0,999	0,990	0,991	0,989	0,728	0,699	0,841	0,645	0,880	0,895	0,929	0,959	0,960	0,969	0,955	0,974	0,987	0,731	0,986	0,942
Acanthamoeba sp. clinical isolate 7	0,992	0,934	0,773	0,858	0,952	0,960	0,962	0,964	0,964	0,963	0,966	0,967	0,980	0,973	0,980	0,999		0,989	0,990	0,988	0,728	0,699	0,841	0,645	0,880	0,895	0,929	0,958	0,959	0,968	0,954	0,974	0,986	0,731	0,986	0,941
Acanthamoeba sp. clinical isolate 2	0,997	0,938	0,773	0,860	0,955	0,963	0,963	0,968	0,966	0,965	0,970	0,971	0,983	0,973	0,980	0,990	0,989		0,998	0,993	0,726	0,698	0,846	0,647	0,881	0,896	0,930	0,962	0,961	0,969	0,954	0,976	0,987	0,732	0,989	0,942
18S A, polyphaga Linc-AP1	0,998	0,937	0,773	0,860	0,956	0,963	0,962	0,968	0,967	0,966	0,970	0,971	0,983	0,972	0,981	0,991	0,990	0,998		0,994	0,727	0,699	0,845	0,648	0,881	0,897	0,931	0,963	0,961	0,969	0,954	0,976	0,988	0,732	0,990	0,942
EF554328.1 Acanthamoeba castellanii strain Neff ATCC 3	0,994	0,936	0,773	0,861	0,956	0,962	0,963	0,967	0,966	0,965	0,973	0,973	0,982	0,971	0,980	0,989	0,988	0,993	0,994		0,726	0,697	0,846	0,648	0,883	0,898	0,930	0,963	0,962	0,972	0,957	0,975	0,986	0,733	0,990	0,941
AF019065.1 Acanthamoeba tubiashi OC15C 18S ribosoma	0,728	0,710	0,605	0,644	0,717	0,721	0,720	0,714	0,726	0,726	0,723	0,725	0,729	0,725	0,722	0,728	0,728	0,726	0,727	0,726		0,816	0,673	0,537	0,692	0,696	0,711	0,719	0,719	0,726	0,714	0,724	0,730	0,583	0,727	0,700
AF019064.1 Acanthamoeba astronyxis Ray Hayes 18S rib	0,699	0,686	0,587	0,620	0,689	0,689	0,688	0,685	0,695	0,695	0,693	0,694	0,700	0,693	0,693	0,699	0,699	0,698	0,699	0,697	0,816		0,647	0,518	0,666	0,665	0,684	0,689	0,689	0,696	0,683	0,693	0,700	0,562	0,698	0,672
AF019067.1 Acanthamoeba culbertsoni Lilly A1 18S ribos	0,845	0,840	0,706	0,739	0,835	0,839	0,841	0,828	0,841	0,841	0,841	0,842	0,842	0,841	0,837	0,841	0,841	0,846	0,845	0,846	0,673	0,647		0,615	0,837	0,844	0,844	0,833	0,834	0,843	0,827	0,851	0,844	0,693	0,845	0,824
U94735.1 Acanthamoeba lenticulata strain E182 18S sma	0,648	0,643	0,554	0,581	0,636	0,644	0,643	0,635	0,644	0,643	0,641	0,642	0,647	0,645	0,641	0,645	0,645	0,647	0,648	0,648	0,537	0,518	0,615		0,631	0,638	0,638	0,638	0,641	0,646	0,634	0,646	0,646	0,547	0,645	0,628
AY026244.1 Acanthamoeba polyphaga strain ATCC30872	0,881	0,868	0,734	0,769	0,869	0,882	0,883	0,868	0,878	0,877	0,876	0,877	0,875	0,878	0,875	0,880	0,880	0,881	0,881	0,883	0,692	0,666	0,837	0,631		0,927	0,881	0,871	0,872	0,880	0,866	0,883	0,881	0,755	0,884	0,857
U07411.1 Acanthamoeba palestinensis Reich ATCC 3087(	0,896	0,881	0,742	0,781	0,882	0,897	0,898	0,880	0,893	0,892	0,891	0,893	0,894	0,894	0,890	0,895	0,895	0,896	0,897	0,898	0,696	0,665	0,844	0,638	0,927		0,890	0,887	0,890	0,897	0,882	0,899	0,894	0,807	0,897	0,870
AF019053.1 Acanthamoeba pearcei 18S ribosomal RNA g	0,932	0,931	0,809	0,811	0,920	0,930	0,931	0,914	0,929	0,928	0,927	0,928	0,926	0,930	0,925	0,929	0,929	0,930	0,931	0,930	0,711	0,684	0,844	0,638	0,881	0,890		0,919	0,917	0,924	0,910	0,937	0,931	0,727	0,932	0,907
AF346662.1 Acanthamoeba triangularis 18S ribosomal RI	0,964	0,925	0,762	0,835	0,950	0,951	0,952	0,938	0,960	0,959	0,948	0,949	0,957	0,953	0,950	0,959	0,958	0,962	0,963	0,963	0,719	0,689	0,833	0,638	0,871	0,887	0,919		0,943	0,954	0,940	0,958	0,960	0,725	0,960	0,925
AY351646.1 Acanthamoeba divionensis strain AA2 18S ri	0,962	0,928	0,764	0,841	0,944	0,949	0,948	0,946	0,956	0,955	0,948	0,948	0,962	0,950	0,955	0,960	0,959	0,961	0,961	0,962	0,719	0,689	0,834	0,641	0,872	0,890	0,917	0,943		0,981	0,970	0,953	0,966	0,726	0,959	0,919
U07417.1 Acanthamoeba royreba Oak Rge ATCC 30884 1	0,970	0,934	0,768	0,848	0,949	0,958	0,960	0,953	0,961	0,960	0,958	0,959	0,965	0,958	0,960	0,969	0,968	0,969	0,969	0,972	0,726	0,696	0,843	0,646	0,880	0,897	0,924	0,954	0,981		0,982	0,963	0,973	0,732	0,968	0,928
AY351647.1 Acanthamoeba mauritaniensis 18S ribosoma	0,955	0,919	0,757	0,835	0,936	0,943	0,946	0,939	0,948	0,947	0,944	0,944	0,952	0,946	0,946	0,955	0,954	0,954	0,954	0,957	0,714	0,683	0,827	0,634	0,866	0,882	0,910	0,940	0,970	0,982		0,949	0,958	0,719	0,954	0,914
U07414.1 Acanthamoeba castellanii Ma ATCC 50370 18S	0,978	0,941	0,779	0,850	0,952	0,958	0,960	0,954	0,962	0,962	0,965	0,965	0,970	0,982	0,968	0,974	0,974	0,976	0,976	0,975	0,724	0,693	0,851	0,646	0,883	0,899	0,937	0,958	0,953	0,963	0,949		0,972	0,735	0,979	0,952
U07406.1 Acanthamoeba rhysodes BCM:0685:116 ATCC	0,988	0,940	0,774	0,862	0,955	0,965	0,963	0,967	0,966	0,965	0,965	0,966	0,979	0,971	0,980	0,987	0,986	0,987	0,988	0,986	0,730	0,700	0,844	0,646	0,881	0,894	0,931	0,960	0,966	0,973	0,958	0,972		0,730	0,984	0,940
18S A. healyi	0,732	0,719	0,762	0,692	0,721	0,734	0,735	0,719	0,729	0,729	0,729	0,729	0,730	0,731	0,726	0,731	0,731	0,732	0,732	0,733	0,583	0,562	0,693	0,547	0,755	0,807	0,727	0,725	0,726	0,732	0,719	0,735	0,730		0,733	0,710
18S A. quina	0,989	0,936	0,774	0,863	0,953	0,964	0,966	0,967	0,962	0,961	0,974	0,974	0,978	0,973	0,986	0,986	0,986	0,989	0,990	0,990	0,727	0,698	0,845	0,645	0,884	0,897	0,932	0,960	0,959	0,968	0,954	0,979	0,984	0,733		0,947
18S A, polyphaga ATCC 30872	0,943	0,909	0,751	0,823	0,920	0,930	0,933	0,921	0,930	0,929	0,933	0,933	0,934	0,943	0,938	0,942	0,941	0,942	0,942	0,941	0,700	0,672	0,824	0,628	0,857	0,870	0,907	0,925	0,919	0,928	0,914	0,952	0,940	0,710	0,947	

Table S4d.

Seq->	A. castellani Neff	A, polyphaga Linc-AP1	A. royreba	A. hatchetti	A. rhysodes	A. mauritaniensis	A. culbertsoni	A. griffini	A. lugdunensis	A. palestinensis	A. polyphaga ATCC 30872	A. stevensoni	A. terricola	A. quina	A. healyi	Acanthamoeba sp. clinical isolate 8	Acanthamoeba sp. clinical isolate 4	A. triangularis	A. lenticulata	A. divionensis	Acanthamoeba sp. clinical isolate 3	Acanthamoeba sp. environmental isolate 1	Acanthamoeba sp. clinical isolate 5	Acanthamoeba sp. environmental isolate 2	Acanthamoeba sp. clinical isolate 6	Acanthamoeba sp. clinical isolate 7	Acanthamoeba sp. environmental isolate 4	Acanthamoeba sp. clinical isolate 1	A. astronyxis
A. castellani Neff		0,998	0,854	0,976	0,994	0,976	1,000	0,895	0,982	1,000	0,976	0,919	0,998	0,982	0,905	0,982	0,974	0,974	0,859	0,687	0,980	0,978	0,974	0,984	0,984	0,982	0,974	0,980	0,687
A, polyphaga Linc-AP1	0,998		0,856	0,974	0,992	0,974	0,998	0,897	0,980	0,998	0,974	0,917	0,996	0,980	0,903	0,980	0,972	0,972	0,857	0,685	0,978	0,976	0,972	0,982	0,982	0,980	0,972	0,978	0,685
A. royreba	0,854	0,856		0,852	0,856	0,850	0,854	0,848	0,852	0,854	0,852	0,842	0,856	0,854	0,840	0,854	0,848	0,844	0,823	0,690	0,852	0,850	0,850	0,856	0,856	0,854	0,848	0,852	0,688
A. hatchetti	0,976	0,974	0,852		0,982	0,972	0,976	0,901	0,974	0,976	0,972	0,917	0,974	0,978	0,907	0,978	0,976	0,976	0,853	0,694	0,976	0,994	0,970	0,980	0,980	0,978	0,976	0,976	0,694
A. rhysodes	0,994	0,992	0,856	0,982		0,982	0,994	0,901	0,988	0,994	0,982	0,925	0,992	0,988	0,907	0,988	0,980	0,980	0,863	0,687	0,986	0,984	0,980	0,990	0,990	0,988	0,980	0,986	0,687
A. mauritaniensis	0,976	0,974	0,850	0,972	0,982		0,976	0,899	0,974	0,976	0,972	0,913	0,974	0,978	0,901	0,978	0,970	0,970	0,859	0,688	0,976	0,974	0,970	0,980	0,980	0,978	0,970	0,976	0,688
A. culbertsoni	1,000	0,998	0,854	0,976	0,994	0,976		0,895	0,982	1,000	0,976	0,919	0,998	0,982	0,905	0,982	0,974	0,974	0,859	0,687	0,980	0,978	0,974	0,984	0,984	0,982	0,974	0,980	0,687
A. griffini	0,895	0,897	0,848	0,901	0,901	0,899	0,895		0,893	0,895	0,893	0,923	0,895	0,895	0,899	0,895	0,893	0,893	0,843	0,673	0,893	0,895	0,895	0,897	0,897	0,895	0,893	0,893	0,673
A. lugdunensis	0,982	0,980	0,852	0,974	0,988	0,974	0,982	0,893		0,982	0,982	0,913	0,980	0,988	0,899	0,988	0,976	0,976	0,853	0,690	0,986	0,976	0,980	0,990	0,990	0,988	0,976	0,986	0,690
A. palestinensis	1,000	0,998	0,854	0,976	0,994	0,976	1,000	0,895	0,982		0,976	0,919	0,998	0,982	0,905	0,982	0,974	0,974	0,859	0,687	0,980	0,978	0,974	0,984	0,984	0,982	0,974	0,980	0,687
A. polyphaga ATCC 30872	0,976	0,974	0,852	0,972	0,982	0,972	0,976	0,893	0,982	0,976		0,913	0,974	0,990	0,899	0,990	0,968	0,968	0,853	0,688	0,988	0,974	0,994	0,992	0,992	0,990	0,968	0,988	0,688
A. stevensoni	0,919	0,917	0,842	0,917	0,925	0,913	0,919	0,923	0,913	0,919	0,913		0,921	0,915	0,925	0,915	0,911	0,911	0,847	0,667	0,913	0,917	0,915	0,917	0,917	0,915	0,911	0,913	0,667
A. terricola	0,998	0,996	0,856	0,974	0,992	0,974	0,998	0,895	0,980	0,998	0,974	0,921		0,980	0,905	0,980	0,972	0,972	0,857	0,685	0,978	0,976	0,972	0,982	0,982	0,980	0,972	0,978	0,685
A. quina	0,982	0,980	0,854	0,978	0,988	0,978	0,982	0,895	0,988	0,982	0,990	0,915	0,980		0,901	1,000	0,974	0,974	0,855	0,692	0,998	0,980	0,988	0,998	0,998	1,000	0,974	0,998	0,692
A. healyi	0,905	0,903	0,840	0,907	0,907	0,901	0,905	0,899	0,899	0,905	0,899	0,925	0,905	0,901		0,901	0,897	0,897	0,855	0,669	0,899	0,905	0,901	0,903	0,903	0,901	0,897	0,899	0,669
Acanthamoeba sp. clinical isolate 8	0,982	0,980	0,854	0,978	0,988	0,978	0,982	0,895	0,988	0,982	0,990	0,915	0,980	1,000	0,901		0,974	0,974	0,855	0,692	0,998	0,980	0,988	0,998	0,998	1,000	0,974	0,998	0,692
Acanthamoeba sp. clinical isolate 4	0,974	0,972	0,848	0,976	0,980	0,970	0,974	0,893	0,976	0,974	0,968	0,911	0,972	0,974	0,897	0,974		0,996	0,859	0,690	0,972	0,978	0,970	0,976	0,976	0,974	1,000	0,972	0,690
A. triangularis	0,974	0,972	0,844	0,976	0,980	0,970	0,974	0,893	0,976	0,974	0,968	0,911	0,972	0,974	0,897	0,974	0,996		0,863	0,688	0,972	0,978	0,970	0,976	0,976	0,974	0,996	0,972	0,688
A. lenticulata	0,859	0,857	0,823	0,853	0,863	0,859	0,859	0,843	0,853	0,859	0,853	0,847	0,857	0,855	0,855	0,855	0,859	0,863		0,681	0,853	0,851	0,851	0,857	0,857	0,855	0,859	0,853	0,682
A. divionensis	0,687	0,685	0,690	0,694	0,687	0,688	0,687	0,673	0,690	0,687	0,688	0,667	0,685	0,692	0,669	0,692	0,690	0,688	0,681		0,690	0,689	0,688	0,690	0,690	0,692	0,690	0,690	0,994
Acanthamoeba sp. clinical isolate 3	0,980	0,978	0,852	0,976	0,986	0,976	0,980	0,893	0,986	0,980	0,988	0,913	0,978	0,998	0,899	0,998	0,972	0,972	0,853	0,690		0,978	0,986	0,996	0,996	0,998	0,972	1,000	0,690
Acanthamoeba sp. environmental isolate 1	0,978	0,976	0,850	0,994	0,984	0,974	0,978	0,895	0,976	0,978	0,974	0,917	0,976	0,980	0,905	0,980	0,978	0,978	0,851	0,689	0,978		0,972	0,982	0,982	0,980	0,978	0,978	0,689
Acanthamoeba sp. clinical isolate 5	0,974	0,972	0,850	0,970	0,980	0,970	0,974	0,895	0,980	0,974	0,994	0,915	0,972	0,988	0,901	0,988	0,970	0,970	0,851	0,688	0,986	0,972		0,990	0,990	0,988	0,970	0,986	0,688
Acanthamoeba sp. environmental isolate 2	0,984	0,982	0,856	0,980	0,990	0,980	0,984	0,897	0,990	0,984	0,992	0,917	0,982	0,998	0,903	0,998	0,976	0,976	0,857	0,690	0,996	0,982	0,990		1,000	0,998	0,976	0,996	0,690
Acanthamoeba sp. clinical isolate 6	0,984	0,982	0,856	0,980	0,990	0,980	0,984	0,897	0,990	0,984	0,992	0,917	0,982	0,998	0,903	0,998	0,976	0,976	0,857	0,690	0,996	0,982	0,990	1,000		0,998	0,976	0,996	0,690
Acanthamoeba sp. clinical isolate 7	0,982	0,980	0,854	0,978	0,988	0,978	0,982	0,895	0,988	0,982	0,990	0,915	0,980	1,000	0,901	1,000	0,974	0,974	0,855	0,692	0,998	0,980	0,988	0,998	0,998		0,974	0,998	0,692
Acanthamoeba sp. environmental isolate 4	0,974	0,972	0,848	0,976	0,980	0,970	0,974	0,893	0,976	0,974	0,968	0,911	0,972	0,974	0,897	0,974	1,000	0,996	0,859	0,690	0,972	0,978	0,970	0,976	0,976	0,974		0,972	0,690
Acanthamoeba sp. clinical isolate 1	0,980	0,978	0,852	0,976	0,986	0,976	0,980	0,893	0,986	0,980	0,988	0,913	0,978	0,998	0,899	0,998	0,972	0,972	0,853	0,690	1,000	0,978	0,986	0,996	0,996	0,998	0,972		0,690
A. astronyxis	0,687	0,685	0,688	0,694	0,687	0,688	0,687	0,673	0,690	0,687	0,688	0,667	0,685	0,692	0,669	0,692	0,690	0,688	0,682	0,994	0,690	0,689	0,688	0,690	0,690	0,692	0,690	0,690	

Table S4e.

Seq->	A. castellani Neff	A. royreba	A. stevensoni	A. lenticulata	A. polyphaga ATCC 30872	A. quina	A. culbertsoni	A. palestinensis	A. rhysodes	A. terricola	A. lugdunensis	A. healyi	A. hatchetti	A. mauritaniensis	A. griffini	Acanthamoeba sp. clinical isolate 3	Acanthamoeba sp. clinical isolate 4	A. divionensis	Acanthamoeba sp. environmental isolate 1	Acanthamoeba sp. clinical isolate 5	Acanthamoeba sp. environmental isolate 2	Acanthamoeba sp. clinical isolate 6	Acanthamoeba sp. clinical isolate 7	Acanthamoeba sp. clinical isolate 8	Acanthamoeba sp. clinical isolate 2	A. triangularis	A. astronyxis	Acanthamoeba sp. environmental isolate 4	Acanthamoeba sp. clinical isolate 1
A. castellani Neff		0,869	0,924	0,845	0,980	0,988	0,980	0,992	0,992	1,000	0,980	0,914	0,973	0,978	0,897	0,983	0,976	0,690	0,980	0,980	0,983	0,988	0,985	0,974	0,969	0,973	0,690	0,973	0,990
A. royreba	0,869		0,851	0,805	0,861	0,869	0,866	0,864	0,871	0,869	0,866	0,854	0,859	0,866	0,852	0,864	0,861	0,680	0,864	0,861	0,864	0,869	0,866	0,856	0,853	0,859	0,680	0,859	0,866
A. stevensoni	0,924	0,851		0,829	0,919	0,926	0,919	0,922	0,931	0,924	0,919	0,912	0,922	0,917	0,915	0,922	0,919	0,662	0,924	0,919	0,922	0,926	0,924	0,913	0,908	0,917	0,662	0,919	0,924
A. lenticulata	0,845	0,805	0,829		0,840	0,845	0,838	0,849	0,849	0,845	0,838	0,833	0,833	0,849	0,809	0,840	0,842	0,662	0,845	0,838	0,841	0,845	0,842	0,832	0,827	0,845	0,662	0,840	0,849
A. polyphaga ATCC 30872	0,980	0,861	0,919	0,840		0,992	0,985	0,973	0,988	0,980	0,985	0,909	0,973	0,978	0,897	0,988	0,976	0,687	0,980	0,995	0,988	0,992	0,990	0,978	0,973	0,973	0,687	0,973	0,976
A. quina	0,988	0,869	0,926	0,845	0,992		0,992	0,980	0,995	0,988	0,992	0,916	0,980	0,985	0,904	0,995	0,983	0,687	0,988	0,992	0,995	1,000	0,997	0,985	0,981	0,980	0,687	0,980	0,983
A. culbertsoni	0,980	0,866	0,919	0,838	0,985	0,992		0,973	0,988	0,980	1,000	0,909	0,973	0,978	0,897	0,988	0,980	0,690	0,980	0,985	0,988	0,992	0,990	0,978	0,973	0,978	0,690	0,978	0,976
A. palestinensis	0,992	0,864	0,922	0,849	0,973	0,980	0,973		0,985	0,992	0,973	0,919	0,966	0,971	0,890	0,976	0,969	0,685	0,973	0,973	0,976	0,980	0,978	0,967	0,962	0,966	0,685	0,966	0,988
A. rhysodes	0,992	0,871	0,931	0,849	0,988	0,995	0,988	0,985		0,992	0,988	0,916	0,980	0,985	0,904	0,990	0,983	0,687	0,988	0,988	0,990	0,995	0,992	0,981	0,976	0,980	0,687	0,980	0,988
A. terricola	1,000	0,869	0,924	0,845	0,980	0,988	0,980	0,992	0,992		0,980	0,914	0,973	0,978	0,897	0,983	0,976	0,690	0,980	0,980	0,983	0,988	0,985	0,974	0,969	0,973	0,690	0,973	0,990
A. lugdunensis	0,980	0,866	0,919	0,838	0,985	0,992	1,000	0,973	0,988	0,980		0,909	0,973	0,978	0,897	0,988	0,980	0,690	0,980	0,985	0,988	0,992	0,990	0,978	0,973	0,978	0,690	0,978	0,976
A. healyi	0,914	0,854	0,912	0,833	0,909	0,916	0,909	0,919	0,916	0,914	0,909		0,916	0,914	0,883	0,912	0,909	0,673	0,914	0,909	0,912	0,916	0,914	0,903	0,898	0,907	0,673	0,907	0,914
A. hatchetti	0,973	0,859	0,922	0,833	0,973	0,980	0,973	0,966	0,980	0,973	0,973	0,916		0,971	0,904	0,976	0,978	0,690	0,988	0,973	0,976	0,980	0,978	0,967	0,962	0,976	0,690	0,976	0,969
A. mauritaniensis	0,978	0,866	0,917	0,849	0,978	0,985	0,978	0,971	0,985	0,978	0,978	0,914	0,971		0,907	0,980	0,976	0,692	0,983	0,978	0,981	0,985	0,983	0,971	0,966	0,973	0,692	0,973	0,973
A. griffini	0,897	0,852	0,915	0,809	0,897	0,904	0,897	0,890	0,904	0,897	0,897	0,883	0,904	0,907		0,900	0,900	0,673	0,902	0,897	0,900	0,904	0,902	0,891	0,886	0,897	0,673	0,900	0,897
Acanthamoeba sp. clinical isolate 3	0,983	0,864	0,922	0,840	0,988	0,995	0,988	0,976	0,990	0,983	0,988	0,912	0,976	0,980	0,900		0,978	0,692	0,983	0,988	0,990	0,995	0,997	0,985	0,981	0,976	0,692	0,976	0,978
Acanthamoeba sp. clinical isolate 4	0,976	0,861	0,919	0,842	0,976	0,983	0,980	0,969	0,983	0,976	0,980	0,909	0,978	0,976	0,900	0,978		0,690	0,980	0,980	0,978	0,983	0,980	0,969	0,964	0,997	0,690	0,997	0,971
A. divionensis	0,690	0,680	0,662	0,662	0,687	0,687	0,690	0,685	0,687	0,690	0,690	0,673	0,690	0,692	0,673	0,692	0,690		0,687	0,690	0,687	0,687	0,690	0,682	0,680	0,687	0,997	0,687	0,680
Acanthamoeba sp. environmental isolate 1	0,980	0,864	0,924	0,845	0,980	0,988	0,980	0,973	0,988	0,980	0,980	0,914	0,988	0,983	0,902	0,983	0,980	0,687		0,980	0,983	0,988	0,985	0,974	0,969	0,978	0,687	0,978	0,976
Acanthamoeba sp. clinical isolate 5	0,980	0,861	0,919	0,838	0,995	0,992	0,985	0,973	0,988	0,980	0,985	0,909	0,973	0,978	0,897	0,988	0,980	0,690	0,980		0,988	0,992	0,990	0,978	0,973	0,978	0,690	0,978	0,976
Acanthamoeba sp. environmental isolate 2	0,983	0,864	0,922	0,841	0,988	0,995	0,988	0,976	0,990	0,983	0,988	0,912	0,976	0,981	0,900	0,990	0,978	0,687	0,983	0,988		0,995	0,992	0,981	0,978	0,976	0,687	0,976	0,978
Acanthamoeba sp. clinical isolate 6	0,988	0,869	0,926	0,845	0,992	1,000	0,992	0,980	0,995	0,988	0,992	0,916	0,980	0,985	0,904	0,995	0,983	0,687	0,988	0,992	0,995		0,997	0,985	0,981	0,980	0,687	0,980	0,983
Acanthamoeba sp. clinical isolate 7	0,985	0,866	0,924	0,842	0,990	0,997	0,990	0,978	0,992	0,985	0,990	0,914	0,978	0,983	0,902	0,997	0,980	0,690	0,985	0,990	0,992	0,997		0,988	0,983	0,978	0,690	0,978	0,980
Acanthamoeba sp. clinical isolate 8	0,974	0,856	0,913	0,832	0,978	0,985	0,978	0,967	0,981	0,974	0,978	0,903	0,967	0,971	0,891	0,985	0,969	0,682	0,974	0,978	0,981	0,985	0,988		0,976	0,967	0,682	0,967	0,969
Acanthamoeba sp. clinical isolate 2	0,969	0,853	0,908	0,827	0,973	0,981	0,973	0,962	0,976	0,969	0,973	0,898	0,962	0,966	0,886	0,981	0,964	0,680	0,969	0,973	0,978	0,981	0,983	0,976		0,962	0,680	0,962	0,964
A. triangularis	0,973	0,859	0,917	0,845	0,973	0,980	0,978	0,966	0,980	0,973	0,978	0,907	0,976	0,973	0,897	0,976	0,997	0,687	0,978	0,978	0,976	0,980	0,978	0,967	0,962		0,687	0,995	0,969
A. astronyxis	0,690	0,680	0,662	0,662	0,687	0,687	0,690	0,685	0,687	0,690	0,690	0,673	0,690	0,692	0,673	0,692	0,690	0,997	0,687	0,690	0,687	0,687	0,690	0,682	0,680	0,687		0,687	0,680
Acanthamoeba sp. environmental isolate 4	0,973	0,859	0,919	0,840	0,973	0,980	0,978	0,966	0,980	0,973	0,978	0,907	0,976	0,973	0,900	0,976	0,997	0,687	0,978	0,978	0,976	0,980	0,978	0,967	0,962	0,995	0,687		0,969
Acanthamoeba sp. clinical isolate 1	0,990	0,866	0,924	0,849	0,976	0,983	0,976	0,988	0,988	0,990	0,976	0,914	0,969	0,973	0,897	0,978	0,971	0,680	0,976	0,976	0,978	0,983	0,980	0,969	0,964	0,969	0,680	0,969	

Table S4f.

TabS4_Chelkha_Colson_SciRep_Apr2019_vL Lig3

Seq->	A. rhysodes	A. polyphaga ATCC 30872	A. castellani Neff	A, polyphaga Linc-AP1	A. royreba	A. healyi	A. palestinensis	A. lenticulata	A. quina	A. terricola	A. culbertsoni	A. stevensoni	A. griffini	A. lugdunensis	A. mauritaniensis	A. hatchetti	A. triangularis	Acanthamoeba sp. clinical isolate 3	Acanthamoeba sp. clinical isolate 8	Acanthamoeba sp. clinical isolate 2	Acanthamoeba sp. clinical isolate 4	A. tubiashi	Acanthamoeba sp. environmental isolate 1	Acanthamoeba sp. clinical isolate 5	Acanthamoeba sp. environmental isolate 3	Acanthamoeba sp. environmental isolate 2	Acanthamoeba sp. clinical isolate 6	Acanthamoeba sp. clinical isolate 7	Acanthamoeba sp. environmental isolate 4	Acanthamoeba sp. clinical isolate 1	A. astronyxis	A. divionensis
A. rhysodes		0,974	0,997	0,801	0,837	0,896	0,994	0,840	0,977	0,991	0,809	0,907	0,983	0,983	0,988	0,983	0,997	0,983	0,988	0,988	0,988	0,986	0,988	0,974	0,988	0,988	0,988	0,988	0,988	0,997	0,654	0,980
A. polyphaga ATCC 30872	0,974		0,977	0,801	0,837	0,890	0,974	0,843	0,963	0,972	0,801	0,893	0,974	0,985	0,969	0,969	0,977	0,980	0,985	0,985	0,969	0,977	0,969	1,000	0,985	0,985	0,985	0,985	0,969	0,977	0,654	0,966
A. castellani Neff	0,997	0,977		0,803	0,840	0,899	0,997	0,843	0,980	0,994	0,812	0,910	0,986	0,985	0,991	0,986	1,000	0,986	0,991	0,991	0,991	0,988	0,991	0,977	0,991	0,991	0,991	0,991	0,991	1,000	0,657	0,983
A, polyphaga Linc-AP1	0,801	0,801	0,803		0,955	0,789	0,801	0,775	0,793	0,799	0,761	0,806	0,793	0,798	0,801	0,798	0,803	0,804	0,803	0,803	0,798	0,796	0,803	0,801	0,803	0,803	0,803	0,803	0,798	0,803	0,599	0,806
A. royreba	0,837	0,837	0,840	0,955		0,829	0,837	0,798	0,830	0,835	0,798	0,843	0,830	0,834	0,837	0,832	0,840	0,840	0,840	0,840	0,834	0,832	0,837	0,837	0,840	0,840	0,840	0,840	0,834	0,840	0,621	0,843
A. healyi	0,896	0,890	0,899	0,789	0,829		0,896	0,831	0,885	0,894	0,798	0,901	0,888	0,896	0,901	0,888	0,899	0,896	0,901	0,901	0,896	0,893	0,893	0,890	0,901	0,901	0,901	0,901	0,896	0,899	0,627	0,893
A. palestinensis	0,994	0,974	0,997	0,801	0,837	0,896		0,843	0,977	0,991	0,809	0,907	0,983	0,983	0,988	0,983	0,997	0,983	0,988	0,988	0,994	0,986	0,988	0,974	0,988	0,988	0,988	0,988	0,994	0,997	0,657	0,980
A. lenticulata	0,840	0,843	0,843	0,775	0,798	0,831	0,843		0,830	0,838	0,781	0,834	0,838	0,840	0,845	0,835	0,843	0,843	0,843	0,843	0,843	0,837	0,837	0,843	0,843	0,843	0,843	0,843	0,843	0,843	0,618	0,851
A. quina	0,977	0,963	0,980	0,793	0,830	0,885	0,977	0,830		0,980	0,796	0,896	0,966	0,974	0,972	0,966	0,980	0,972	0,977	0,977	0,972	0,975	0,972	0,963	0,977	0,977	0,977	0,977	0,972	0,980	0,642	0,963
A. terricola	0,991	0,972	0,994	0,799	0,835	0,894	0,991	0,838	0,980		0,807	0,905	0,983	0,980	0,986	0,980	0,994	0,980	0,986	0,986	0,986	0,983	0,986	0,972	0,986	0,986	0,986	0,986	0,986	0,994	0,653	0,977
A. culbertsoni	0,809	0,801	0,812	0,761	0,798	0,798	0,809	0,781	0,796	0,807		0,806	0,802	0,801	0,803	0,804	0,812	0,801	0,803	0,803	0,803	0,804	0,809	0,801	0,803	0,803	0,803	0,803	0,803	0,812	0,607	0,798
A. stevensoni	0,907	0,893	0,910	0,806	0,843	0,901	0,907	0,834	0,896	0,905	0,806		0,902	0,896	0,913	0,899	0,910	0,899	0,901	0,901	0,901	0,899	0,904	0,893	0,901	0,901	0,901	0,901	0,901	0,910	0,613	0,910
A. griffini	0,983	0,974	0,986	0,793	0,830	0,888	0,983	0,838	0,966	0,983	0,802	0,902		0,977	0,977	0,972	0,986	0,977	0,983	0,983	0,977	0,980	0,977	0,974	0,983	0,983	0,983	0,983	0,977	0,986	0,648	0,969
A. lugdunensis	0,983	0,985	0,985	0,798	0,834	0,896	0,983	0,840	0,974	0,980	0,801	0,896	0,977		0,977	0,977	0,985	0,988	0,994	0,994	0,977	0,986	0,977	0,985	0,994	0,994	0,994	0,994	0,977	0,985	0,660	0,969
A. mauritaniensis	0,988	0,969	0,991	0,801	0,837	0,901	0,988	0,845	0,972	0,986	0,803	0,913	0,977	0,977		0,977	0,991	0,977	0,983	0,983	0,983	0,980	0,983	0,969	0,983	0,983	0,983	0,983	0,983	0,991	0,649	0,991
A. hatchetti	0,983	0,969	0,986	0,798	0,832	0,888	0,983	0,835	0,966	0,980	0,804	0,899	0,972	0,977	0,977		0,986	0,972	0,977	0,977	0,977	0,974	0,994	0,969	0,977	0,977	0,977	0,977	0,977	0,986	0,652	0,969
A. triangularis	0,997	0,977	1,000	0,803	0,840	0,899	0,997	0,843	0,980	0,994	0,812	0,910	0,986	0,985	0,991	0,986		0,986	0,991	0,991	0,991	0,988	0,991	0,977	0,991	0,991	0,991	0,991	0,991	1,000	0,657	0,983
Acanthamoeba sp. clinical isolate 3	0,983	0,980	0,986	0,804	0,840	0,896	0,983	0,843	0,972	0,980	0,801	0,899	0,977	0,988	0,977	0,972	0,986		0,994	0,994	0,977	0,986	0,977	0,980	0,994	0,994	0,994	0,994	0,977	0,986	0,652	0,969
Acanthamoeba sp. clinical isolate 8	0,988	0,985	0,991	0,803	0,840	0,901	0,988	0,843	0,977	0,986	0,803	0,901	0,983	0,994	0,983	0,977	0,991	0,994		1,000	0,983	0,991	0,983	0,985	1,000	1,000	1,000	1,000	0,983	0,991	0,654	0,974
Acanthamoeba sp. clinical isolate 2	0,988	0,985	0,991	0,803	0,840	0,901	0,988	0,843	0,977	0,986	0,803	0,901	0,983	0,994	0,983	0,977	0,991	0,994	1,000		0,983	0,991	0,983	0,985	1,000	1,000	1,000	1,000	0,983	0,991	0,654	0,974
Acanthamoeba sp. clinical isolate 4	0,988	0,969	0,991	0,798	0,834	0,896	0,994	0,843	0,972	0,986	0,803	0,901	0,977	0,977	0,983	0,977	0,991	0,977	0,983	0,983		0,980	0,983	0,969	0,983	0,983	0,983	0,983	1,000	0,991	0,657	0,974
A. tubiashi	0,986	0,977	0,988	0,796	0,832	0,893	0,986	0,837	0,975	0,983	0,804	0,899	0,980	0,986	0,980	0,974	0,988	0,986	0,991	0,991	0,980		0,980	0,977	0,991	0,991	0,991	0,991	0,980	0,988	0,652	0,972
Acanthamoeba sp. environmental isolate 1	0,988	0,969	0,991	0,803	0,837	0,893	0,988	0,837	0,972	0,986	0,809	0,904	0,977	0,977	0,983	0,994	0,991	0,977	0,983	0,983	0,983	0,980		0,969	0,983	0,983	0,983	0,983	0,983	0,991	0,651	0,974
Acanthamoeba sp. clinical isolate 5	0,974	1,000	0,977	0,801	0,837	0,890	0,974	0,843	0,963	0,972	0,801	0,893	0,974	0,985	0,969	0,969	0,977	0,980	0,985	0,985	0,969	0,977	0,969		0,985	0,985	0,985	0,985	0,969	0,977	0,654	0,966
Acanthamoeba sp. environmental isolate 3	0,988	0,985	0,991	0,803	0,840	0,901	0,988	0,843	0,977	0,986	0,803	0,901	0,983	0,994	0,983	0,977	0,991	0,994	1,000	1,000	0,983	0,991	0,983	0,985		1,000	1,000	1,000	0,983	0,991	0,654	0,974
Acanthamoeba sp. environmental isolate 2	0,988	0,985	0,991	0,803	0,840	0,901	0,988	0,843	0,977	0,986	0,803	0,901	0,983	0,994	0,983	0,977	0,991	0,994	1,000	1,000	0,983	0,991	0,983	0,985	1,000		1,000	1,000	0,983	0,991	0,654	0,974
Acanthamoeba sp. clinical isolate 6	0,988	0,985	0,991	0,803	0,840	0,901	0,988	0,843	0,977	0,986	0,803	0,901	0,983	0,994	0,983	0,977	0,991	0,994	1,000	1,000	0,983	0,991	0,983	0,985	1,000	1,000		1,000	0,983	0,991	0,654	0,974
Acanthamoeba sp. clinical isolate 7	0,988	0,985	0,991	0,803	0,840	0,901	0,988	0,843	0,977	0,986	0,803	0,901	0,983	0,994	0,983	0,977	0,991	0,994	1,000	1,000	0,983	0,991	0,983	0,985	1,000	1,000	1,000		0,983	0,991	0,654	0,974
Acanthamoeba sp. environmental isolate 4	0,988	0,969	0,991	0,798	0,834	0,896	0,994	0,843	0,972	0,986	0,803	0,901	0,977	0,977	0,983	0,977	0,991	0,977	0,983	0,983	1,000	0,980	0,983	0,969	0,983	0,983	0,983	0,983		0,991	0,657	0,974
Acanthamoeba sp. clinical isolate 1	0,997	0,977	1,000	0,803	0,840	0,899	0,997	0,843	0,980	0,994	0,812	0,910	0,986	0,985	0,991	0,986	1,000	0,986	0,991	0,991	0,991	0,988	0,991	0,977	0,991	0,991	0,991	0,991	0,991		0,657	0,983
A. astronyxis	0,654	0,654	0,657	0,599	0,621	0,627	0,657	0,618	0,642	0,653	0,607	0,613	0,648	0,660	0,649	0,652	0,657	0,652	0,654	0,654	0,657	0,652	0,651	0,654	0,654	0,654	0,654	0,654	0,657	0,657		0,649
A. divionensis	0,980	0,966	0,983	0,806	0,843	0,893	0,980	0,851	0,963	0,977	0,798	0,910	0,969	0,969	0,991	0,969	0,983	0,969	0,974	0,974	0,974	0,972	0,974	0,966	0,974	0,974	0,974	0,974	0,974	0,983	0,649	

Table S4g.

Seq->	A. castellani Neff	A. royreba	A. hatchetti	A. rhysodes	A. mauritaniensis	A. culbertsoni	A. griffini	A. lugdunensis	A. palestinensis	A. polyphaga ATCC 30872	A. stevensoni	A. terricola	A. quina	A. healyi	Acanthamoeba sp. clinical isolate 8	Acanthamoeba sp. clinical isolate 4	A. triangularis	A. lenticulata	A. divionensis	Acanthamoeba sp. clinical isolate 3	Acanthamoeba sp. environmental isolate 1	Acanthamoeba sp. clinical isolate 5	Acanthamoeba sp. environmental isolate 2	Acanthamoeba sp. clinical isolate 6	Acanthamoeba sp. clinical isolate 7	Acanthamoeba sp. environmental isolate 4	Acanthamoeba sp. clinical isolate 1	A. astronyxis	NC_007089.4_919657-922497_Dictyostelium_discoideum_AX4_Alanine_tRNA_ligase
A. castellani Neff		0,855	0,978	0,994	0,981	0,941	0,921	0,982	0,996	0,978	0,919	0,997	0,982	0,906	0,982	0,979	0,981	0,850	0,770	0,982	0,982	0,977	0,986	0,987	0,985	0,978	0,989	0,674	0,319
A. royreba	0,855		0,849	0,856	0,852	0,842	0,844	0,852	0,852	0,851	0,846	0,854	0,851	0,842	0,851	0,849	0,848	0,810	0,729	0,853	0,851	0,850	0,854	0,856	0,854	0,848	0,853	0,657	0,305
A. hatchetti	0,978	0,849		0,982	0,973	0,927	0,922	0,975	0,975	0,972	0,914	0,975	0,975	0,905	0,974	0,977	0,979	0,841	0,769	0,975	0,992	0,971	0,978	0,979	0,978	0,976	0,976	0,676	0,317
A. rhysodes	0,994	0,856	0,982		0,985	0,940	0,925	0,986	0,991	0,982	0,923	0,992	0,986	0,907	0,986	0,983	0,985	0,852	0,768	0,986	0,986	0,981	0,989	0,991	0,989	0,982	0,989	0,673	0,317
A. mauritaniensis	0,981	0,852	0,973	0,985		0,929	0,923	0,976	0,978	0,973	0,915	0,979	0,978	0,905	0,977	0,975	0,977	0,852	0,774	0,978	0,979	0,972	0,981	0,982	0,981	0,975	0,979	0,674	0,317
A. culbertsoni	0,941	0,842	0,927	0,940	0,929		0,870	0,937	0,938	0,930	0,889	0,939	0,933	0,877	0,931	0,929	0,930	0,830	0,718	0,933	0,932	0,929	0,935	0,936	0,935	0,928	0,932	0,658	0,314
A. griffini	0,921	0,844	0,922	0,925	0,923	0,870		0,918	0,918	0,917	0,915	0,920	0,917	0,891	0,918	0,919	0,920	0,831	0,755	0,919	0,920	0,918	0,922	0,923	0,922	0,919	0,920	0,661	0,315
A. lugdunensis	0,982	0,852	0,975	0,986	0,976	0,937	0,918		0,979	0,984	0,911	0,980	0,985	0,901	0,986	0,978	0,979	0,844	0,767	0,987	0,978	0,983	0,990	0,992	0,990	0,977	0,982	0,676	0,318
A. palestinensis	0,996	0,852	0,975	0,991	0,978	0,938	0,918	0,979		0,975	0,917	0,994	0,979	0,907	0,979	0,978	0,978	0,851	0,767	0,979	0,979	0,974	0,982	0,984	0,982	0,977	0,987	0,673	0,317
A. polyphaga ATCC 30872	0,978	0,851	0,972	0,982	0,973	0,930	0,917	0,984	0,975		0,910	0,975	0,982	0,900	0,985	0,971	0,972	0,846	0,765	0,986	0,975	0,996	0,989	0,990	0,989	0,970	0,981	0,674	0,318
A. stevensoni	0,919	0,846	0,914	0,923	0,915	0,889	0,915	0,911	0,917	0,910		0,918	0,914	0,914	0,911	0,912	0,913	0,838	0,733	0,913	0,916	0,911	0,915	0,917	0,915	0,912	0,917	0,646	0,318
A. terricola	0,997	0,854	0,975	0,992	0,979	0,939	0,920	0,980	0,994	0,975	0,918		0,982	0,905	0,979	0,977	0,979	0,847	0,768	0,980	0,980	0,975	0,983	0,985	0,983	0,976	0,986	0,673	0,318
A. quina	0,982	0,851	0,975	0,986	0,978	0,933	0,917	0,985	0,979	0,982	0,914	0,982		0,901	0,988	0,975	0,977	0,844	0,765	0,989	0,979	0,982	0,990	0,992	0,992	0,975	0,987	0,672	0,317
A. healyi	0,906	0,842	0,905	0,907	0,905	0,877	0,891	0,901	0,907	0,900	0,914	0,905	0,901		0,902	0,901	0,901	0,841	0,732	0,902	0,905	0,901	0,905	0,907	0,905	0,900	0,904	0,651	0,314
Acanthamoeba sp. clinical isolate 8	0,982	0,851	0,974	0,986	0,977	0,931	0,918	0,986	0,979	0,985	0,911	0,979	0,988	0,902		0,975	0,976	0,844	0,767	0,993	0,979	0,984	0,993	0,994	0,996	0,974	0,986	0,673	0,317
Acanthamoeba sp. clinical isolate 4	0,979	0,849	0,977	0,983	0,975	0,929	0,919	0,978	0,978	0,971	0,912	0,977	0,975	0,901	0,975		0,995	0,849	0,769	0,975	0,980	0,973	0,979	0,980	0,978	0,999	0,977	0,676	0,317
A. triangularis	0,981	0,848	0,979	0,985	0,977	0,930	0,920	0,979	0,978	0,972	0,913	0,979	0,977	0,901	0,976	0,995		0,851	0,770	0,977	0,982	0,975	0,980	0,982	0,980	0,994	0,978	0,674	0,318
A. lenticulata	0,850	0,810	0,841	0,852	0,852	0,830	0,831	0,844	0,851	0,846	0,838	0,847	0,844	0,841	0,844	0,849	0,851		0,720	0,846	0,845	0,844	0,847	0,849	0,847	0,848	0,849	0,651	0,303
A. divionensis	0,770	0,729	0,769	0,768	0,774	0,718	0,755	0,767	0,767	0,765	0,733	0,768	0,765	0,732	0,767	0,769	0,770	0,720		0,768	0,768	0,766	0,768	0,768	0,770	0,768	0,768	0,892	0,308
Acanthamoeba sp. clinical isolate 3	0,982	0,853	0,975	0,986	0,978	0,933	0,919	0,987	0,979	0,986	0,913	0,980	0,989	0,902	0,993	0,975	0,977	0,846	0,768		0,979	0,985	0,993	0,995	0,996	0,975	0,989	0,674	0,318
Acanthamoeba sp. environmental isolate 1	0,982	0,851	0,992	0,986	0,979	0,932	0,920	0,978	0,979	0,975	0,916	0,980	0,979	0,905	0,979	0,980	0,982	0,845	0,768	0,979		0,974	0,982	0,984	0,982	0,979	0,981	0,673	0,318
Acanthamoeba sp. clinical isolate 5	0,977	0,850	0,971	0,981	0,972	0,929	0,918	0,983	0,974	0,996	0,911	0,975	0,982	0,901	0,984	0,973	0,975	0,844	0,766	0,985	0,974		0,988	0,989	0,988	0,972	0,980	0,674	0,319
Acanthamoeba sp. environmental isolate 2	0,986	0,854	0,978	0,989	0,981	0,935	0,922	0,990	0,982	0,989	0,915	0,983	0,990	0,905	0,993	0,979	0,980	0,847	0,768	0,993	0,982	0,988		0,998	0,996	0,978	0,989	0,674	0,317
Acanthamoeba sp. clinical isolate 6	0,987	0,856	0,979	0,991	0,982	0,936	0,923	0,992	0,984	0,990	0,917	0,985	0,992	0,907	0,994	0,980	0,982	0,849	0,768	0,995	0,984	0,989	0,998		0,998	0,979	0,990	0,674	0,317
Acanthamoeba sp. clinical isolate 7	0,985	0,854	0,978	0,989	0,981	0,935	0,922	0,990	0,982	0,989	0,915	0,983	0,992	0,905	0,996	0,978	0,980	0,847	0,770	0,996	0,982	0,988	0,996	0,998		0,978	0,990	0,675	0,317
Acanthamoeba sp. environmental isolate 4	0,978	0,848	0,976	0,982	0,975	0,928	0,919	0,977	0,977	0,970	0,912	0,976	0,975	0,900	0,974	0,999	0,994	0,848	0,768	0,975	0,979	0,972	0,978	0,979	0,978		0,976	0,675	0,317
Acanthamoeba sp. clinical isolate 1	0,989	0,853	0,976	0,989	0,979	0,932	0,920	0,982	0,987	0,981	0,917	0,986	0,987	0,904	0,986	0,977	0,978	0,849	0,768	0,989	0,981	0,980	0,989	0,990	0,990	0,976		0,673	0,318
A. astronyxis	0,674	0,657	0,676	0,673	0,674	0,658	0,661	0,676	0,673	0,674	0,646	0,673	0,672	0,651	0,673	0,676	0,674	0,651	0,892	0,674	0,673	0,674	0,674	0,674	0,675	0,675	0,673		0,301
NC_007089.4_919657-922497_Dictyostelium_discoideum_A	0,319	0,305	0,317	0,317	0,317	0,314	0,315	0,318	0,317	0,318	0,318	0,318	0,317	0,314	0,317	0,317	0,318	0,303	0,308	0,318	0,318	0,319	0,317	0,317	0,317	0,317	0,318	0,301	
